# Pax 6 Controls Neural Crest Potential of Limbal Niche Cells to Support Self-Renewal of Limbal Epithelial Stem Cells

**DOI:** 10.1038/s41598-019-45100-7

**Published:** 2019-07-05

**Authors:** Szu-Yu Chen, Anny M. S. Cheng, Yuan Zhang, Ying-Ting Zhu, Hua He, Megha Mahabole, Scheffer C. G. Tseng

**Affiliations:** 1 R&D Department, Tissue Tech, Inc., Miami, FL 33126 USA; 2 Department of Biochemistry and Molecular Biology, University of Miami Miller School of Medicine, Miami, FL33136 USA; 3 Department of Ophthalmology, Florida International University, Herbert Wertheim College of Medicine, Miami, FL33199 USA; 4 Ocular Surface Center, and Ocular Surface Research & Education Foundation, Miami, FL 33126 USA

**Keywords:** Stem-cell niche, Stem-cell research

## Abstract

On ocular surface, corneal epithelial stem cells (SC) reside in limbus between cornea and conjunctiva. Pax6, an evolutionally conserved transcription factor essential for eye development, is expressed in post-natal corneal and limbal epithelia progenitors (LEPC) but not in underlying stroma. Because Pax6 is transiently expressed in developing corneal stroma and a subset of limbal and corneal stromal progenitors, we examined the role of Pax6 in limbal niche cells (LNC) in maintaining the phenotype of neural crest (NC) progenitors to support LEPC. Our results showed that nuclear Pax6 staining was found in freshly isolated LNC but not corneal stromal cells. Serial passaged LNC resulted in gradual loss of nuclear Pax6 (46 kDa) staining and neural crest progenitor status defined by the expression of embryonic SCs and NC markers, neurosphere formation, and differentiation into neurons, oligodendrocytes and astrocytes. Gain of function of 46 kDa Pax6 in late-passaged LNC resulted in nuclear Pax6 staining and promotion of the aforementioned NC progenitor status. In an *in vitro* reunion assay, early passaged LNC and late passaged LNC with overexpression of Pax6 inhibited the expression of corneal epithelial differentiation marker and promoted holoclone by LEPC. Therefore, expression of nuclear 46 kDa Pax6 in LNC plays an important developmental role in maintaining NC progenitor status to support self-renewal of corneal epithelial SCs in the limbal niche.

## Introduction

On the ocular surface, corneal epithelial stem cells (SC) reside in the limbus bordered between the cornea and the conjunctiva^1^. From the limbal stroma subjacent to limbal epithelial SC, we have isolated and expanded a subpopulation of limbal niche cells (LNC) that express SC markers such as Oct4, Sox2, Nanog, Rex1, Nestin, N-cadherin and SSEA4^2,3^ and exhibit differentiation potential into vascular endothelial cells, pericytes, osteoblasts, chondrocytes, and adipocytes^4^. From the entire human limbal stroma, others have also isolated progenitors that can differentiate into neurons^5^ and retinal sensory cilia^6^. We^3^ and other^7^ have reported that limbal niche cells (LNC) in the stroma support limbal epithelial stem (progenitor) cells better by promoting holoclones and preventing corneal epithelial differentiation than that in central corneal stromal cells. Interestingly, a subpopulation of corneal stromal cells (CSC) can also be isolated to exhibit sphere formation and differentiation potential into adipocytes^8^, neurons^8,9^, and chondrocytes^8^ besides keratocan-expressing keratocytes^9–12^. The aforementioned limbal and corneal stromal progenitors express developmental neural crest genes, such as ATP binding cassette (ABCG2)^13^, Nestin^9^, Musashi-1^8^, Sox2^6^, Six2/3^11^, and Sox9^8^. These results indicate that both limbal and corneal stroma may contain multi-potent progenitors. It is plausible that these stromal progenitors are derived from migrating peri-ocular mesenchyme of the cranial neural crest during development^14,15^.

Paired box homeotic gene 6 (Pax6) is an evolutionally conserved transcription factor essential for proper development of eye, central nerve system, craniofacial skeletal, olfactory epithelium, and pancreas (Review in^16^). In the eye, the primarily function of Pax6 is mediated the commitment of head ectoderm of optic vesicle into the lens ectoderm and promote the formation of lens vesicle^17^. Homozygous Pax6-deficient mouse embryo exhibits lack of eyes and nose and dies soon at birth^18,19^. Expression of Pax6 is dosage dependent as mutation or missing allele leads to aniridia in humans (Review in^16^) and the small eye (sey, Pax6^+/−^) in mouse animal model^20^. Patient with aniridia-related keratopathy (ARK) observed as typical ocular surface disease with limbal stem cells deficiency (LSCD). However, the pathophysiology of underlying mechanism that lead to LSCD remains to be elucidated. Post-natal expression of Pax6 is restricted to corneal and limbal epithelial cells^21^. Studies reported inadequate levels of Pax6 in corneal epidermis leads to abnormal differentiation in human^22^ and mouse^21^. Interestingly, Pax6^+/−^ in heterozygous adult mice has profound severe defect in cornea stroma and endothelium but less of impact in epithelial cells with delay wound healing^23,24^. Because transient expression of Pax6 is noted in the corneal stroma during development (Review in^25^) and in aforementioned limbal and corneal stromal progenitors^11^, we hypothesize that expression of Pax6 in the limbal stroma might have a unique developmental role in maintaining corneal epithelial homeostasis. Herein, we found the expression and nuclear localization of Pax6 differentiates LNC from CSC and causally correlates with the neural crest progenitor status regarding marker expression, neurosphere formation, and neuroglial differentiation. Furthermore, such a phenotype is crucial to endow LNC with the capability of supporting self-renewal of limbal epithelial SCs by suppressing corneal epithelial differentiation and maintaining holoclone formation.

## Results

### Unique nuclear expression of 46 kDa Pax6 in limbal niche cells

**Figure 1:**
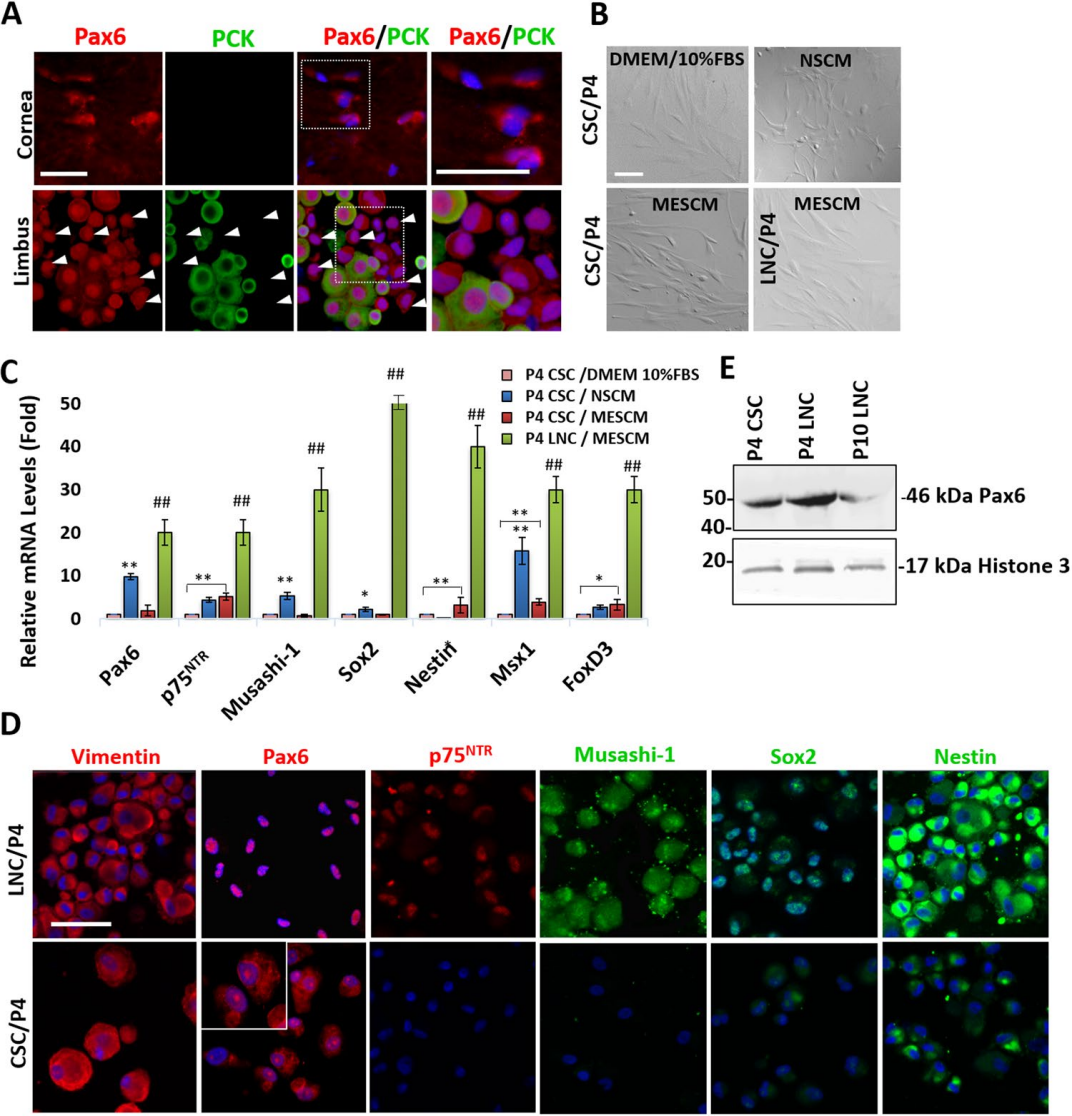
Unique Nuclear 46 kDa Pax6 in Limbal Niche Cells (LNC). Freshly isolated PCK (−) LNC (arrows) and PCK (+) limbal epithelial cells from the limbal tissue exhibited positive nuclear staining of Pax6 while freshly isolated PCK (−) CSC from epithelially denuded corneal stroma exhibited cytoplasmic staining of Pax6 (A). LNC and CSC were expanded in the same manner on coated Matrigel in MESCM up to passage 4 (P4) while CSC were also cultured on plastic in neural stem cell medium (NSCM) or DMEM/10% FBS. Comparison was made on cell morphology by phase microscopy on day 6 (**B**). Transcript expression by RT-qPCR of neural crest markers (Pax6, p75^NTR^, Musashi-1, Sox2, Nestin, Msx1, and FoxD3) in P4 LNC was compared to that of P4 CSC under the identical culture condition (**C**, ^##^p < 0.05, n = 3). P4 CSC in MESCM was further compared to P4 CSC expanded in NSCM using the control P4 CSC in DMEM/10%FBS set as 1 (**C**, *p < 0.1; **p < 0.05, n = 3). Immunofluorescence staining showed the cytolocalization of vimentin (red), Pax6 (red), p75^NTR^ (red), Musashi-1 (green), Sox2 (green) and Nestin (green) in P4 LNC and P4 CSC on coated Matrigel in MESCM (**D**, nuclear counterstaining by Hoechst 33342). Scale bars = 100 µm in (**A**,**B**,**D**). Protein expression of Pax6 from P4 CSC, P4 LNC and P10 LNC were confirmed by Western blot using Histone 3 as a loading control (**E**).

To determine whether there was any difference between LNC and CSC in the expression of Pax6 immediately after isolation, we isolated them from epithelium-containing limbal stroma and epithelially denuded corneal stroma from the same donor using collagenase digestion as reported^2^. Double immunostaining of Pax6 and pan-cytokeratin (PCK) showed positive nuclear staining of Pax6 in PCK (+) epithelial cells as expected but also in freshly isolated PCK (−) LNC (Fig. 1A, arrows). In contrast, weak cytoplasmic staining of Pax6 was noted in PCK (−) CSC (Fig. 1A). LNC and CSC were then expanded on coated Matrigel in a modified serum-free ESC medium (MESCM)^26^ and compared to CSC expanded on plastic in DMEM/10%FBS^27^ or in neural stem cell expansion medium (NSCM)^12,28^. Phase images showed that cells in these cultures at the same passage 4 (P4) all exhibited similar spindle cells (Fig. 1B). Compared to P4 CSC cultured on coated Matrigel in MESCM, P4 LNC had significant higher transcript expression of Pax6 as well as other neural crest markers such as p75^NTR^, Musashi-1, Sox2, Nestin, Msx1, and FoxD3 (Fig. 1C, ^##^p < 0.05). Compared to P4 CSC expanded on coated Matrigel in MESCM, expression of Pax6, Musashi-1, Sox2 and Msx1 was higher in P4 CSC cultured in NSCM (Fig. 1C, *p < 0.1, **p < 0.05), but the expression of p75^NTR^, Nestin, Msx1, and FoxD3 transcripts was downregulated when p4 CSC were cultured in DMEM/10%FBS (Fig. 1C, *p < 0.1, **p < 0.05). Immunofluorescence staining confirmed the universal expression of vimentin by these mesenchymal cells. However, nuclear staining of Pax6 was noted in P4 LNC while cytoplasmic staining of Pax6 was predominantly noted in P4 CSC when both cultured on coated Matrigel in MESCM (Fig. 1D). In addition, P4 LNC expressed nuclear expression of p75^NTR^, Musashi-1, Sox2, and Sox10 (Supplementary Fig. S1) and strong cytoplasmic expression of Nestin. In contrast, the CSC counterpart expressed weak or absent with the nuclear staining of Pax6, p75^NTR^, Musashi-1, and Sox2 and exhibited weak cytoplasmic staining of Nestin (Fig. 1D). After confirming the specificity of the antibody to recognize 46 kDa Pax6 protein in the positive control of ARPE-19 cell lysate as previously reported^29,30^ (Supplementary Fig. S5B), we then demonstrated by western blot analysis that 46 kDa Pax6 protein was prominently expressed by P4 LNC more so than P4 CSC (Fig. 1E and Supplementary Fig. S5B). These results collectively suggested that 46 kDa Pax6 contributed to the nuclear Pax6 staining of P4 LNC and correlated with high expressions of other neural crest markers.

### Nuclear Pax6 in LNC is lost after serial passage

**Figure 2:**
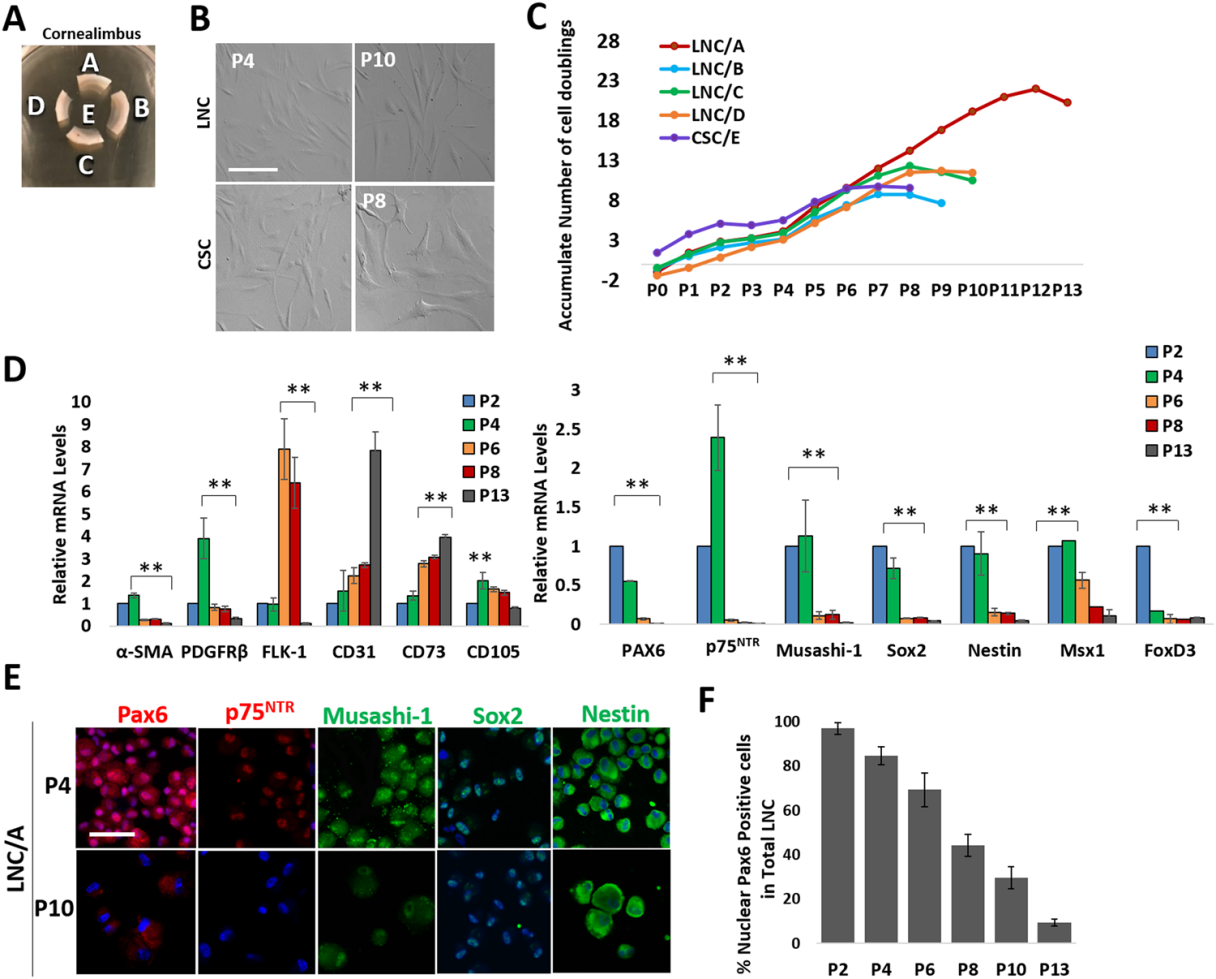
Loss of Nuclear Pax6 Staining in LNC after Serial Passages. LNC and CSC were isolated from four quadrants (labeled as **A**–**D**) and central cornea (labeled as **E**) of the same donor (A) and were serially passaged to measure cumulative doubling time (**C**) on coated Matrigel in MESCM. Comparison on cell morphology was determined by phase microscopy on day 6 (**B**) and transcript expression of angiogenic markers (α-SMA, PDGFRβ, FLK-1, CD31), mesenchymal stem cells markers (CD73 and CD105) (**D**), and neural crest markers (Pax6, p75^NTR^, Musashi-1, Sox2, Nestin, FoxD3 and Msx1) were determined by RT-qPCR using the transcript expression level of each marker in P2 set at 1 (**D**, **p < 0.01, n = 3). Immunofluorescence staining showed the cytolocalization of Pax6 (red), p75^NTR^ (red), Musashi-1 (green), Sox2 (green), and Nestin (green) (**E**, nuclear counterstaining by Hoechst 33342). The percentage of cells with nuclear Pax6 staining in total LNC from region A was declined during the serial passages (**F**). Scale bar = 100 µm in (**B**,**E**).

We have previously reported that P4 LNC exhibit vascular angiogenic potential to differentiate into vascular endothelial cells or pericytes capable of stabilizing vascular tube formation^4^ and more potent potential than human bone marrow-derived mesenchymal stem cells to differentiate into osteoblasts, chondrocytes, and adipocytes^4^. To know whether serial passages might affect the aforementioned nuclear localization of Pax6 and expression of the aforementioned neural crest markers in LNC, we isolated LNC from four different limbal quadrants (labeled as A – D) and CSC from the central cornea (labeled as E) of the same donor tissue (Fig. 2A) and serially expanded them on coated Matrigel in MESCM in a similar manner as reported^26^. Both LNC and CSC exhibited similar spindle cells at P4 and gradual cell enlargement at P10 (Fig. 2B). LNC from Region A (i.e., the superior limbus) reached P13 with 20.2 cumulative cell doublings, LNC from Regions B - D reached P8 - P9 with an average of 10.9 ± 1.9 cumulative cell doublings, while CSC reached P8 with 9.6 cell doublings (Fig. 2C). Consistent with our previous report^26^, LNC expanded after P2 did not express transcripts of such epithelial markers as cytokeratin 12 (CK12) and cytokeratin 15 (CK15). (Supplementary Fig. S2). Also consistent with our previous reports^3,4^, transcript expression of pericyte markers such as α-SMA, PDGFRβ, and mesenchymal stem cell markers such as CD105 was higher at P4. Herein, we further noted the continuous expression of FLK-1(VEGFR2), CD31, and CD73 by serial passage (Fig. 2D, **p < 0.01, n = 3). Compared to the expression level at P4, serial passages reduced expression of Pax6, p75^NTR^, Musashi-1, Sox2, Nestin, FoxD3 and Msx1 in LNC isolated from Region A (Fig. 2D, **p < 0.01, n = 3) and Region B (Supplementary Fig. S3). Immunofluorescence staining further showed that nuclear staining of Pax6 by P4 LNC and became nearly nil staining by P10 LNC; nuclear staining of p75^NTR^ and Sox2 at P4 was lost in P10 LNC (Fig. 2E). Cytoplasmic and nuclear staining of Musashi-1 and Nestin at P4 was reduced at P10 when cell enlargement was noted (Fig. 2E). Western Blot analysis confirmed that 46 kDa Pax6 protein was prominently expressed by P4 LNC and nearly nil in P10 LNC. (Fig. 1E and Supplementary Fig. S5B) The percentage of nuclear Pax6 (+) LNC in Region A showed a progressive decline by serial passages (Fig. 2F). These data collectively indicated that serial passage of LNC on coated Matrigel in MESCM resulted in the progressive loss of nuclear Pax6, which was accompanied by decreased expression of neural crest markers and increased expression of angiogenesis and MSC markers.

### Neural potential in LNC declines by serial passage

**Figure 3:**
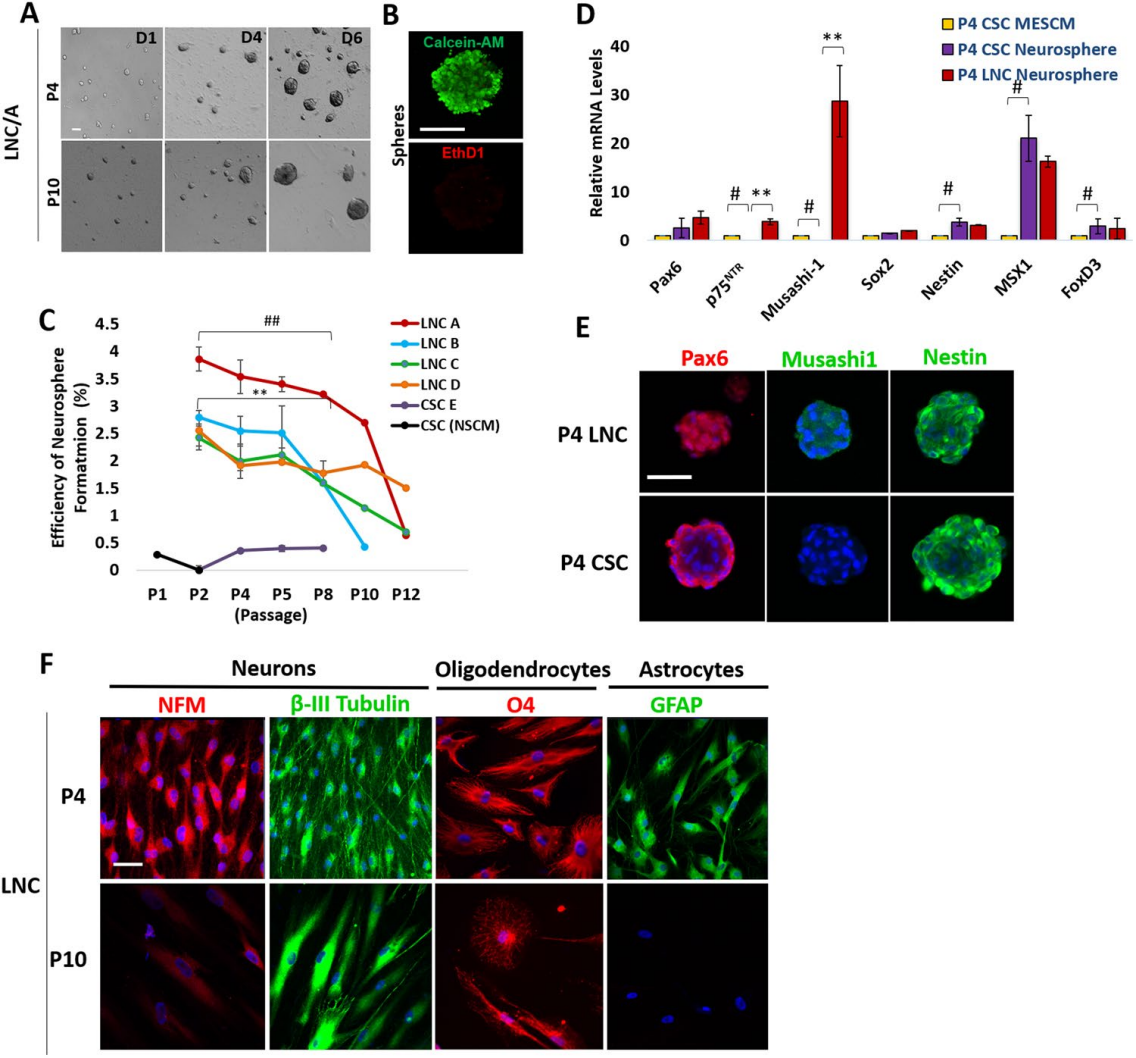
Neural Potential of LNC and CSC Declines after Serial Passages. For each passage, 5 × 10^3^/cm^2^ LNC cells were seeded on 12 well plate coated with poly-HEMA in NSCM neurosphere medium to generate neurospheres for 6 days (**A**, representative images from P4 and P10). Live and dead assay showed the sphere formed by P4 LNC was alive on day 6 (green) without dead cells (red) (**B**). The neurosphere-forming efficiency (%) was measured from LNC expanded from four different limbal regions and was compared with that of CSC region at each passage (**C**, ^##^p = 0.001; **p = 0.001). The transcript level of neural crest markers such as Pax6, p75^NTR^, Musashi-1, Sox2, Nestin, Msx1, and FoxD3 in neurospheres formed by P4 CSC was compared with those by P4 LNC or P4 CSC seeded on coated Matrigel in MESCM which the transcript expression was set as 1 (**D**, **p = 0.0001; ^#^p = 0.001, n = 3, respectively). Immunofluorescence staining showed cytolocalization of Pax6 (red), Musashi-1 (green) and Nestin (green) in neurospheres derived from P4 CSC and P4 LNC (**E**). P4 or P10 LNC were assessed for their potential of differentiation into neurons, oligodendrocytes, and astrocytes by immunofluorescence staining of neurofilament M (NFM, red) and β-III tubulin (green), O_4_ (red), and Glial fibrillary acidic protein (GFAP, green), respectively (**F**). Nuclear counterstaining by Hoechst 33342 in (**E**,**F**). Scale bars = 50 µm in (**A**,**F**), 200 µm in (**B**), and 100 µm in (**E**).

*In vitro* neurosphere growth assay is gold standard for neural stem cells^31^. Because serial passage of LNC led to reduced expression and loss of nuclear Pax6 staining and other neural crest markers, we wondered whether such a change was correlated with the loss of the neural progenitor status defined by neurosphere formation and neuroglial differentiation potential. LNC from 4 regions and CSC were serially passaged and seeded at the same density of 5 × 10^3^/cm^2^ in poly-HEMA coated 12-well in the neurosphere medium containing 1.6% of methylcellulose for 7 days^32^. Spheres emerged with an increasing size (Fig. 3A, representative P4 and P10 LNC from Region A). Live and dead assay showed these spheres from P4 LNC on day 6 were alive as shown by positive calcein-AM staining (green) and negative ethidium homodimer staining (red) (Fig. 3B). The counting of spheres with a size of greater than 50 µm in diameter at day 6 showed that CSC yielded a very low sphere-forming efficiency, i.e., 0.3 ± 0.1%, between P2 to P8 (Fig. 3C). In contrast, P2 LNC from all 4 regions had a significant higher efficiency of 2.9 ± 0.5% (^##^p = 0.0006, n = 3) with Region A being significantly higher than other 3 regions (Fig. 3C, **p = 0.003, n = 3). For all limbal regions, the sphere-forming efficiency declined after serial passage and reached 0.8 ± 0.4% by P10 (Fig. 3C). P4 LNC neuropheres expressed a significantly higher transcript level of p75^NTR^ and Musashi-1 than P4 CSC neurospheres (Fig. 3D, **p = 0.001, n = 3). P4 CSC neurospheres expressed significantly lower levels of p75^NTR^ and Musashi-1 (Fig. 3D, ^#^p = 0.001, n = 3) but higher levels of Nestin and Msx1 (Fig. 3D, ^#^p = 0.001, n = 3) than P4 CSC cultured on coated Matrigel as the control. Immunofluorescence staining confirmed the positive nuclear Pax6 staining and cytoplasmic and nuclear staining of Musashi-1 in P4 LNC neurospheres but weak cytoplasmic staining of Pax6 and negative expression of Musashi-1 in P4 CSC neurospheres, and no difference in the staining pattern of Nestin (Fig. 3E). P4 LNC cultured on coated Matrigel could be differentiated into neurons with expression of neurofilament M (NFM, red) and β-III tubulin (green), oligodendrocytes with expression of O_4_, and astrocytes with expression of glial fibrillary acidic protein (GFAP) (Fig. 3F). As a comparison, P10 LNC could not differentiate into astrocytes although they were still able to adopt differentiation into neurons and oligodendrocytes with larger cells (Fig. 3F). These results collectively supported the notion that serial passage of LNC resulted in the loss of the neural crest progenitor status as evidenced by reduced neurosphere formation and neuroglial differentiation potential.

### Forced expression of Pax6 restores neural crest progenitor status in P10 LNC

**Figure 4:**
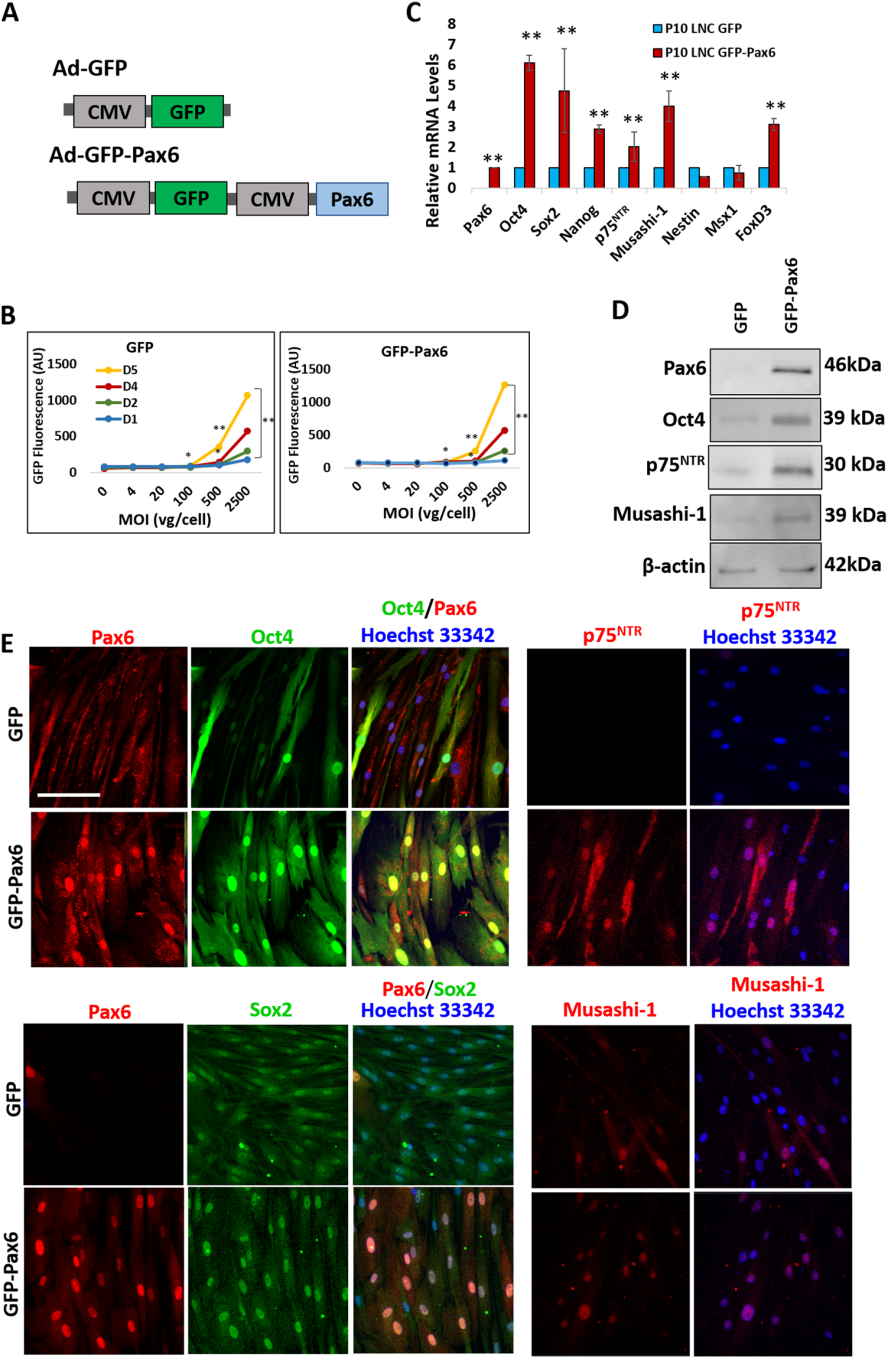
Forced Expression of Pax6 Upregulates Expression of Neural Crest Markers in P10 LNC. Transfection of Ad-GFP (GFP) or Ad-GFP-Pax6 (GFP-Pax6) plasmid (**A**) was performed in P10 LNC cultured on coated Matrigel in MESCM after their respective multiplicity of infection (MOI) was pre-determined during a period of 5 days (**B**, *p < 0.1, **p < 0.05, n = 3). Following the respective transfection, RT-PCR analysis was used to compare the transcript levels of ESC markers (Oct4, Sox2, and Nanog) and neural crest markers (p75^NTR^, Musashi-1, Nestin, Msx1, and FoxD3) (**C**, **p < 0.05, n = 3). Western blot analysis was used to compare the protein expression of 46 kDa Pax6, Oct4, p75^NTR^, and Musashi-1 using β-actin as the loading control (**D**). Cytolocalization of Pax6 (red) and Oct4 (green), Pax6 (red) and Sox2 (green), as well as p75^NTR^ (red) and Musashi-1 (red) (**E**) were determined by either double or single immunofluorescence staining. Nuclear counterstaining by Hoechst 33342. Scale bar = 100 µm.

We then explored the causal relationship by forced expression of Pax6 in P10 LNC, which exhibited a gradual loss of transcript expression of Oct4, Sox2, Nanog, and Rex1^4^ and the loss of nuclear Pax6 staining as well as expression of neural crest markers (Fig. 2). We first confirmed the optimal transfection efficiency of the adenoviral plasmid construct with CMV promoter and enhanced green fluorescent protein (GFP) with or without Pax6, i.e., Ad-GFP-Pax6 (experimental) and Ad-GFP (control) (Fig. 4A) to be at the multiplicity of infection (MOI) of 100 (Fig. 4B, *p < 0.1 and **p < 0.05, n = 3). P10 LNC transfected by GFP-Pax6 upregulated transcript expression of ESC markers (Oct4, Sox2, Nanog) and neural crest markers (p75^NTR^, Musashi-1, and FoxD3) when compared to cells transfected by GFP (Fig. 4C, **p < 0.05, n = 3). Western blot analysis showed overexpression in P10 LNC enhanced the intensity of 46 kDa Pax6 band (Fig. 4D). Following the overexpression of 46 kDa Pax6, there was upregulation of Oct4 (39 kDa), p75^NTR^ (30 kDa), and Musashi-1 (39 kDa) proteins (Fig. 4D). Immunofluorescence staining confirmed nuclear Pax6 staining (red) in P10 LNC transfected by GFP-Pax6 but not GFP (Fig. 4E). Nuclear Pax6 staining was co-localized with enhanced nuclear staining of Oct4 and Sox2 (Fig. 4E). In addition, forced expression of Pax6 also resulted in enhanced nuclear and cytoplasmic expression of p75^NTR^ and nuclear expression of Musashi-1 (Fig. 4E).

**Figure 5:**
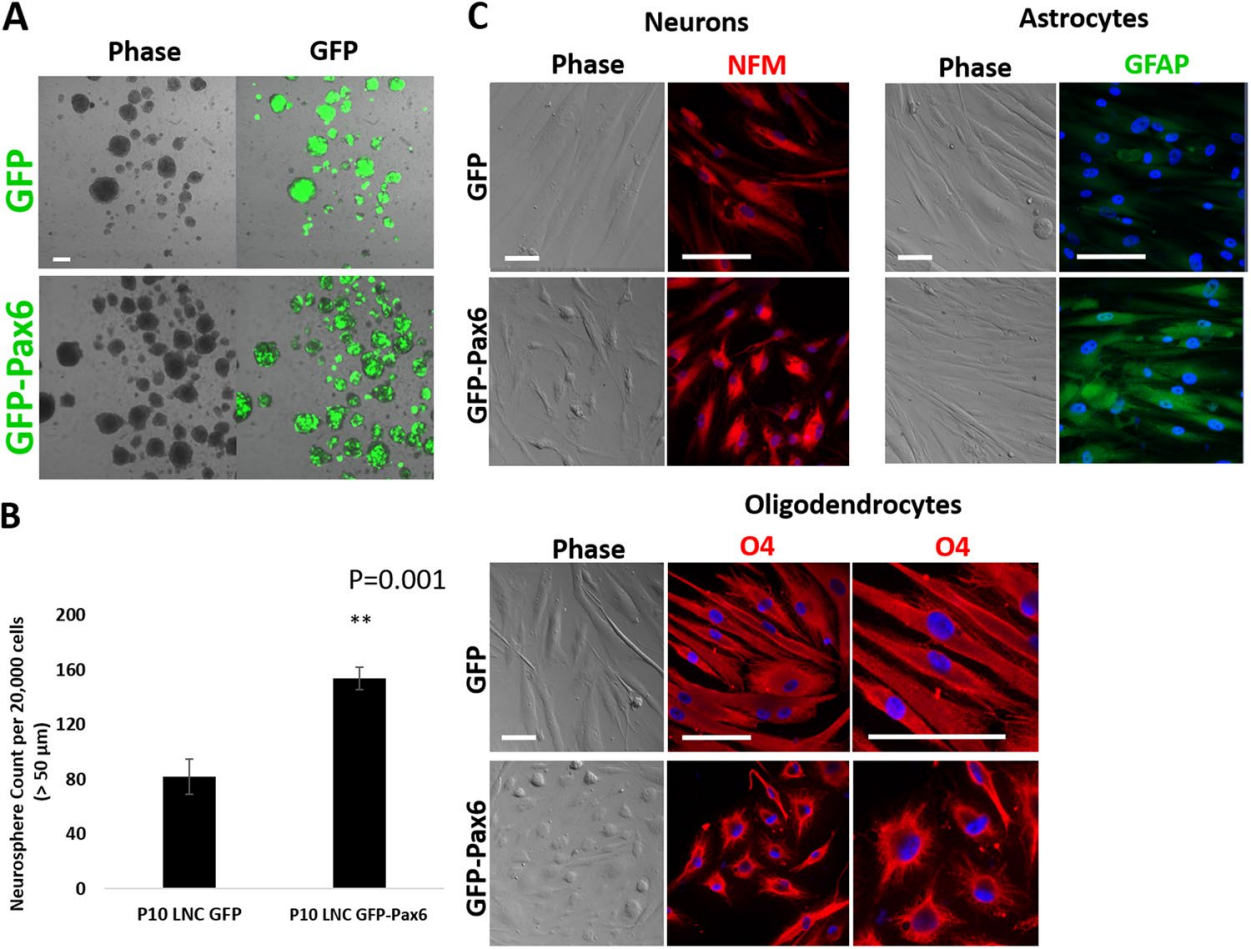
Forced Expression of Pax6 Restores Neural Crest Progenitor Status in P10 LNC. P10 LNC on coated Matrigel in MESCM was transfected with Ad-GFP (GFP) or Ad-GFP-Pax6 (GFP-Pax6) plasmid at MOI 100 for 4 days, then the medium was switched to NSCM neurosphere medium for 7 days. Neurospheres were imaged by confocal microscopy with or without fluorescence for GFP (**A**). The total numbers of neurospheres with a size greater than 50 μm in diameter were compared (**B**, *p = 0.001, n = 3). The differentiation potential for cells derived from neurospheres was assessed after cells were cultured in different induction media and observed by phase microscopy and immunofluorescence staining of neurofilament M (NFM, red), O_4_ (red), and glial fibrillary acidic protein (GFAP, green) (**C**, nuclear counterstaining by Hoechst 33342, scale bars = 50 µm).

Neurosphere formation (Fig. 5A) and neurosphere-forming efficiency (Fig. 5B, n = 3) were also significantly promoted in P10 LNC transfected by GFP-Pax6 when compared to cells transfected by GFP. Furthermore, cell morphology was reduced in size in P10 LNC transfected by GFP-Pax6 during their respective differentiation into neuronal, astrocytes and oligodendrocytes (Fig. 5C). The loss of differentiation potential into astrocyte by P10 LNC (Fig. 3F) was restored after transfection with GFP-Pax6, which also promoted the potential to differentiate into neurons with strong expression of NFM and oligodendrocytes with expression of O_4_ (Fig. 5C). These data collectively indicated a strong causal relationship between the nuclear localization of Pax6 and the restoration of the neural crest progenitor status.

### P10 LNC with forced expression of Pax6 supports self-renewal of LEPC

**Figure 6:**
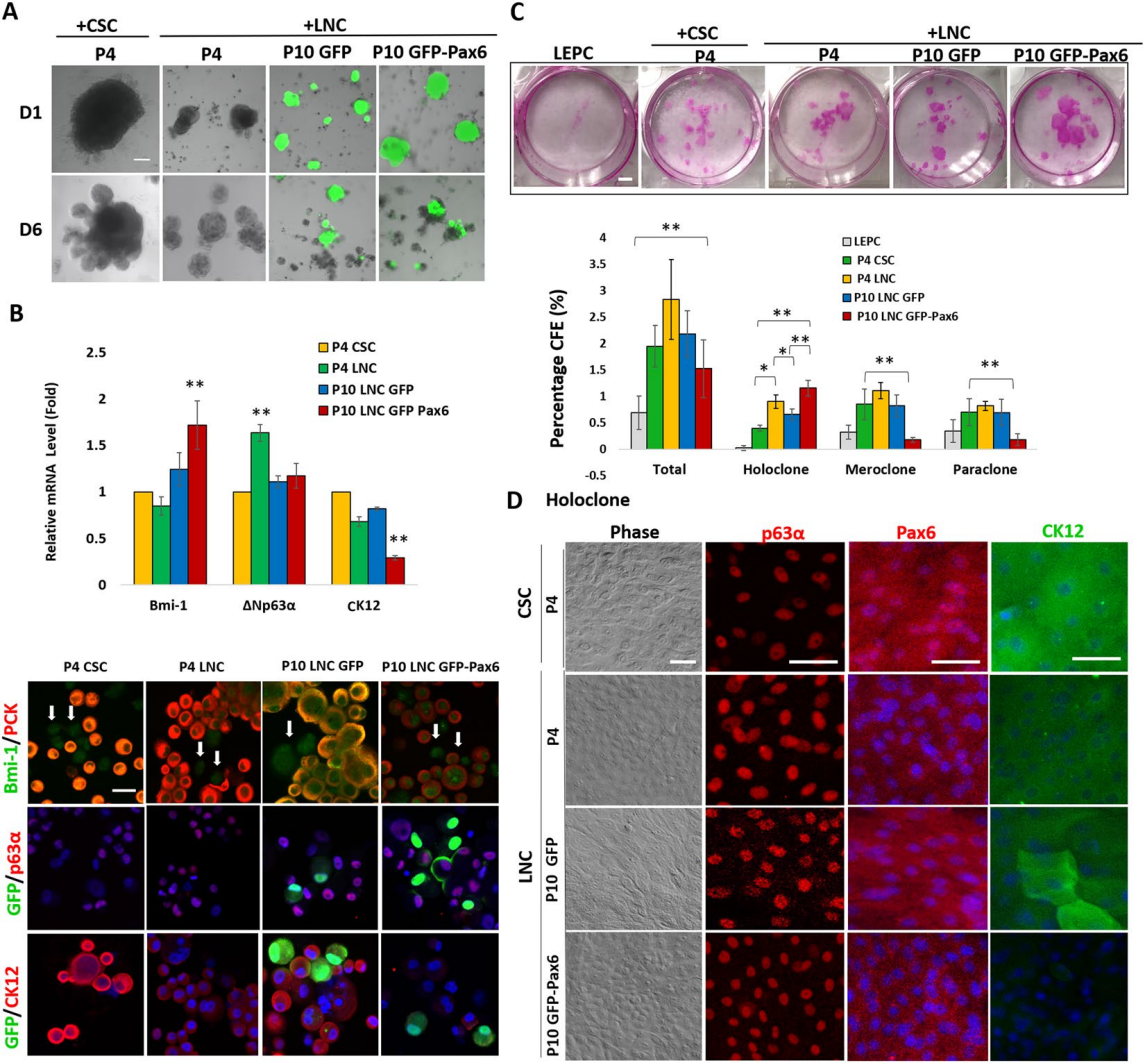
P10 LNC with Forced Expression of Pax6 Promotes Self-renewal of LEPC. *In vitro* reunion assay was performed between P10 LNC transfected with Ad-GFP or Ad-GFP-Pax6 plasmid at MOI 100 and LEPC in comparison with the positive control of P4 LNC and the negative control of P4 CSC. Sphere morphology was imaged by phase and GFP fluorescence under confocal microscopy at Day 1 and Day 6 (**A**). The resultant reunion spheres were analyzed by qRT-PCR for transcript expression of Bmi-1 (**p = 0.003, n = 3), ∆Np63α (**p = 0.06, n = 3), and cytokeratin 12 (CK12) (**p = 0.000004, n = 3) when compared with P4 CSC as the control (**B**). Double immunostaining was performed for Bmi-1 (green)/PCK (red), GFP/p63α (red), and GFP/CK12 (red) for PCK (+) cells. (B, white arrows indicate PCK (−) cells). *In vitro* clonal assay for LEPC with or without reunion with P10 LNC transfected with Ad-GFP or Ad-GFP-Pax6, P4 LNC or P4 CSC was performed on 3T3 fibroblast feeder layers. The clonal growth was assessed by rhodamine B staining while the colony-forming efficiency (%) for total, holoclone, meroclone, and paraclone was compared (**C**, *p < 0.05; ** < 0.01). The epithelial morphology of holoclone was further characterized by phase image and immunostaining of p63α, Pax6, and CK12 (**D**). Nuclear counterstaining by Hoechst 33342. Scale bars = 50 µm in (**A**,**B**, and **D**) = 0.5 mm in (**C**).

Reunion of single LEPC with single P4 LNC^26^ or P4 LNC aggregates^33^ in 3D Matrigel prevents corneal fate decision/differentiation of limbal epithelial progenitor cells (LEPC). Furthermore, corneal fate decision is prevented more by reunion between LEPC and P4 LNC than that between LEPC and P4 CSC^4^. Herein, we repeated the same experiment and noted that reunion between LEPC and P4 LNC generated similar cell aggregates (Fig. 6A) but with higher expression of ∆Νp63α and reduced expression of CK12 when compared to that between LEPC and P4 CSC (Fig. 6B). Under the same condition, reunion of LEPC with P10 LNC did not alter the transcript expression but promoted expression of CK12 protein when compared to that with P4 LNC (Fig. 6B), suggesting that loss of nuclear Pax6 staining in P10 LNC was associated with the outcome favorable of driving LEPC toward more corneal fate decision. In contrast, compared to that with P10 LNC, reunion with P10 LNC with forced expression of Pax6 significantly higher transcript expression of Bmi1 but downregulated CK12 transcript and protein (Fig. 6B), suggesting that gain of Pax6 expression in LNC was linked to suppression of corneal fate decision in LEPC.

In an *in vitro* colony forming assay on mitomycin-treated 3T3 fibroblast feeder layers, reunion between LEPC and P4 LNC on 3D Matrigel yielded greater clonal growth of holoclone^34^. Herein, we noted that the colony-forming efficiency (CFE) of holoclone was significantly promoted when reunion of LEPC was made with P4 LNC when compared to LEPC alone or with P4 CSC (Fig. 6C, *p = 0.02) when the same number of PCK+ cells were seeded. Compared to reunion between LEPC and P4 LNC, the CFE of holoclone was significantly reduced in reunion between LEPC and P10 LNC-GFP (Fig. 6C, *p = 0.02), suggesting that late passage LNC, which lost nuclear Pax6 staining (Fig. 2), did not support clonal growth of LEPC as potent as P4 LNC, which maintained nuclear Pax6 staining (Fig. 2). In contrast, the holoclone CFE was significantly promoted in reunion between LEPC and P10 LNC with forced expression of Pax6 when compared to that between LEPC and P10 LNC GFP (Fig. 6C, **p = 0.0001), suggesting that nuclear Pax6 staining (Fig. 3) endowed P10 LNC with a capacity of supporting clonal growth by LEPC. Further characterization of the resultant holoclone by immunofluorescence staining revealed nuclear p63α+ holoclone in LEPC no matter if they were reunioned with P4 CSC, P4 LNC, or P10 LNC with or without forced expression of Pax6 (Fig. 6D). However, nuclear Pax6+ LEPC were noted in holoclone formed following reunion with P4 CSC, both nuclear Pax6+ and Pax6− LEPC were noted in holoclone formed following reunion with P4 LNC and P10 LNC GFP, while nuclear Pax6- LEPCs were noted in holoclone formed following reunion with P10 LNC GFP-Pax6 (Fig. 6D). CK12+ basal and suprabasal LEPC were noted in holoclone generated following reunion with P4 CSC and P10 LNC GFP, CK12+ basal LEPC were noted in holoclone generated following reunion with P4 LNC, while CK12- basal LEPC were noted in holoclone generated following reunion with P10 LNC GFP-Pax6 (Fig. 6D). Collectively, these findings strongly suggested that overexpression of Pax6 in P10 LNC prevented corneal fate decision and promoted holoclone formation by LEPC in 3D Matrigel.

## Discussion

During eye morphogenesis, Pax6-expressing cranial neural crest cells are involved in the formation of lens placodes^35^, retina^36^, and anterior segment^37^. During eye development, nuclear Pax6+ staining is observed in corneal stroma^37,38^, ciliary body^37^, endothelial^38^ and trabecular meshwork^38^. Herein, we found nuclear Pax6+ staining in freshly isolated (Fig. 1) and early passaged (P4) of LNC (Fig. 2), but not in their corneal counterpart, i.e., P4 CSC, which exhibited weak cytoplasmic Pax6 staining (Fig. 1). Western blot analysis confirmed that it was 46 kDa Pax6 responsible for the nuclear Pax6 staining of P4 LNC (Fig. 1). Because such a phenotype was correlated with higher expression of ESC markers such as Oct4, Sox2 and many other neural crest markers such as p75^NTR^, Musashi-1, Sox2, Nestin, Msx1 and FoxD3 (Fig. 2), neurosphere formation (Fig. 3) and differentiation potential into neuroglial lineages (Fig. 3), we surmise that nuclear staining with 46 kDa Pax6 in LNC is a hallmark to signify the neural crest progenitor status. The role of Pax6 in neuronal differentiation has also been reported by others. The strong nuclear Pax6+ staining has also been noted in radial glia cells of the ventricular (germinal) zone housing neural stem/progenitor cells^39^. Pax6-haploinsufficiency leads to reduced production of neural stem/progenitors in adult hippocampus rat^40^. Non-viral plasmid transfection of Pax6 and Sox2 in adult human fibroblast direct reprogram cells to a neural precursor cell-like state^41,42^. The Pax6 -Brg1/BAF complex is essential and sufficient to convert glia into neuron in the adult mouse olfactory bulb^43^. Hence, a gradual loss of nuclear Pax6 staining in LNC during serial passage might contribute to the gradual loss of the expression of neural crest markers and reduction of neurosphere formation and neuroglial differentiation potential (Fig. 3). Interestingly, such gradual loss of neural crest potential during serial passage was correlated with an increase expression of angiogenesis and MSC markers (Fig. 3), suggesting that LNC have the plasticity of undergoing both neuronal and vascular differentiation potentials, a notion that has also been noted in adult mammalian neural crest derived carotid body^44^. Future studies are needed to see if LNC can be ascribed an important role in partaking in regenerative wound healing, which requires restoration of both neural and vascular tissue components.

The critical role of Pax6 in governing the neural crest progenitor status was further substantiated by forced expression of 46 kDa Pax6 in late passaged LNC. Gain of function by forced expression with adenoviral vector GFP-Pax6 resulted in the reappearance of nuclear 46 kDa Pax6 staining in P10 LNC and re-expression of neural crest markers (Fig. 4) and increased neurosphere formation and neuroglial differentiation potential (Fig. 5). We already learned from previous studies that expression of ESC markers such as Oct4, Sox2, Nanog and Rex1, which are noted in freshly isolated LNC^2^ is also gradually lost during serial passage^4^. Herein, we noted that forced expression of Pax6 in P10 LNC helped regain expression of Oct4 and Sox2 and neural crest markers (Fig. 5). Chromatin immunoprecipitation chip sequencing study reveals that Pax6 targets to several gene promotors in neural progenitor cells^45^ Pax6 binds directly to pluripotent genes, Oct4 and Nanog to repress their expression and to promote neural neuroectoderm genes in human ES cells^46^, and cooperates with Sox2 to ensure the unidirectional lineage commitment toward neuronal differentiation in radial glial cells^47^. Therefore, it is plausible that nuclear localization of Pax6 might help to reinforce the nuclear Oct4, Sox2, and Nanog to ensure the neural crest progenitor status in LNC.

One should not be mixed the nuclear Pax6 staining in LNC reported here with that is known to express in post-natal corneal and limbal epithelia^21,22^. For the latter, Pax6 together with p63 specifies limbal epithelial SCs from the surface ectoderm and with Wnt7A controls corneal fate decision by promoting CK12 expression by limbal and corneal epithelial cells^48^. To demonstrate the important role of Pax6 in LNC to modulate self-renewal of limbal epithelial SCs, we utilized an *in vitro* colony forming assay, which is frequently used to measure the self-renewal property of a single SC. For epithelial stem (progenitor) cells, the standard of proof relies on categorizing resultant clones based on morphology and phenotypic characterization as holoclone, meroclone, and paraclone^49^. Only holoclones are capable of performing extensive proliferation and self-renewal, whilst meroclones have a limited proliferative capacity and cannot self-renew and paraclones are incapable of further proliferation^49,50^. Previously, we followed the aforementioned practice, adopted the same criteria, and reported that the reunion of P4 LNC with limbal epithelial progenitor cells (LEPC) supports self-renewal of the latter in 3D Matrigel by demonstrating the greater yield of holoclones with nil expression of corneal epithelial differentiation marker, cytokeratin 12, when compare to LEPC alone. (Li *et al*. 2012) Herein, by taking advantage of our success in establishing the *in vitro* reunion assay between LNC and LEPC, which contain limbal epithelial SCs^34^, we showed that P10 LNC, which lost nuclear Pax6 staining (Fig. 2), yielded fewer holoclones than P4 LNC (Fig. 6C). In contrast, reunion between LEPC and P10 LNC with forced expression of Pax6 yielded significantly more holoclones than LEPC alone or reunion between P10 LNC GFP and LEPC (Fig. 6C*)*. Consistent with our previous findings^3,26^, we showed the reunion between LEPC and P4 LNC prevents corneal fate decision as evidenced by suppression of CK12 expression and promoted holoclone formation in LEPC when compared to LEPC alone or LEPC with P4 CSC (Fig. 6B). Although transcript expression of epithelial progenitor markers such as Bmi-1^51^ and ∆Νp63α^52^ and corneal fate maker such as CK12 did not change in LEPC when reunion with P4 LNC or P10 LNC, forced expression of 46 kDa Pax6 in P10 LNC upregulated Bmi-1 transcript and downregulated CK12 transcript and protein (Fig. 6B), indicating that Pax6 plays an important role in LNC in preventing LEPC from taking corneal fate decision. This finding was accompanied by an increase of CFE of holoclone (Fig. 6C), in which the basal epithelial monolayer uniquely exhibited small uniform nuclear p63α+ staining, Pax6- nuclear staining, and negative CK12 (Fig. 6D).

We thus conclude that Pax6 plays an important role in LNC to support self-renewal of limbal epithelial SC. The finding that LNC from the superior limbus, i.e., Region A, maintained the longest passage number with the highest nuclear Pax6+ staining and exhibited greatest neurosphere formation (Fig. 2) also supports the general belief that superior limbus contains the most prominent limbal palisade of Vogt, which specifies the limbal SC niche^53^. Expression of Pax6 is known to dosage dependent manners (Fukuda *et al*. 2000, Sansome *et al*. 2009), further studies to define the signaling mechanism that governs nuclear location of Pax6 in LNC using neural crest-restricted Pax6 conditional knockout should help us understand why mutation or missing allele of Pax6 leads to limbal SC deficiency in human patients with aniridia^54,55^.

## Material and Methods

### Cell isolation and expansion

Human corneolimbal rim and central cornea button stored at 4 °C in Optisol (Chiron Vision, Irvine, CA) for less than 7 days were obtained from different donors (Florida Lions Eye Bank, Miami, FL). After rinsing three times with PBS pH 7.4 containing 50 µg/ml gentamicin and 1.25 µg/ml amphotericin B, the excess sclera, conjunctiva, iris, corneal endothelium and trabecular meshwork were removed up to the Schwalbe’s line for the corneoscleral rim before being cut into superior, nasal, inferior, and temporal quadrants (Fig. 2A, denoted as region A to D) at 1 mm within and beyond the anatomic limbus. As report previously^56^, an intact epithelial sheet including basal epithelial cells was obtained by subjecting each limbal quadrant to digestion with 10 mg/ml dispase in modified embryonic stem cell medium (MESCM), which was made of Dulbecco’s Modified Eagle’s Medium (DMEM)/F-12 nutrient mixture (F-12) (1:1) supplemented with 10% knockout serum, 10 ng/ml LIF, 4 ng/ml bFGF, 5 mg/ml insulin, 5 mg/ml transferrin, 5 ng/ml sodium selenite supplement (ITS), 50 µg/ml gentamicin and 1.25 µg/ml amphotericin B in plastic dishes containing at 4 °C for 16 h under humidified 5% CO_2_ incubator. LNC were isolated by digestion with 2 mg/ml collagenase A at 37 °C for 16 h to generate floating clusters^2,26^. CSC were isolated in the same manner except that the overlying epithelium from the central cornea (Fig. 2A, denoted as region E) was digested with 10 mg/ml of dispase II at 37 °C for 2 h in MESCM to remove epithelial sheets first.

For expansion, single cells derived from limbal clusters or CSC after digestion with 0.25% trypsin and 1 mM EDTA (T/E) were seeded at 1 × 10^4^/cm^2^ in the 6-well plate pre-coated with 5% Matrigel in MESCM and cultured in humidified 5% CO_2_ with media change every 3–4 days for total 6–7 days. In some instance, CSC were expanded in Neural Stem Cells Serum-Free Expansion Medium (NSCM) consist of DMEM/F-12 (1:1) supplemented, 2% Neural Supplement (consist of B-27 and N-2), 20 ng/ml human FGF-basic recombinant, 20 ng/ml human EGF recombinant. CSC were also expanded on plastic in DMEM with 10% FBS, 50 µg/ml gentamicin and 1.25 µg/ml amphotericin B. When cells reach at 80–90% confluence and were serially expanded at the seeding density of 5 × 10^3^ per cm^2^ for up to 13 passages. The extent of total expansion was measured by the number of cell doubling (NCD) calculate from formulate: NCD = log10(y/x)/log10^2^, where “y” is the final density of the cells and “x” is the initial seeding density of the cells. All material used for cell isolation and cell culture expansion are listed in Supplementary Table S1.

#### *In vitro* reunion assay

*In vitro* reunion assay was performed as reported^26^. In brief, P4 LNC, P4 CSC, and P10 LNC transfected with Ad-GFP or Ad-GFP-Pax6 that were expanded on coated Matrigel were seeded in 3D Matrigel at the density of 5 × 10^4^ cells/cm^2^ to generate aggregates in MESCM for 24 h. Single LEPC obtained from dispase-isolated limbal epithelial sheet were seeded at the density of 5 × 10^4^ cells/cm^2^ in 3D Matrigel with or without the aforementioned LNC or CSC aggregates for 6 days. The resultant spheres were harvested by digesting Matrigel with 10 mg/ml dispase II at 37 °C for 2 h, of which some were rendered into single cells by T/E before being prepared for cytospin.

#### *In vitro* colony forming assay

An *in vitro* epithelial colony forming assay was performed as reported^2^ on mitomycin-treated 3T3 fibroblast feeder layers in supplemental hormonal epithelial medium (SHEM), which was made of an equal volume of HEPES-buffered DMEM and Ham’s F-12 containing bicarbonate, 0.5% dimethyl sulfoxide, 2 ng/ml mouse-derived epidermal growth factor, 5 mg/ml insulin, 5 mg/ml transferrin, 5 ng/ml sodium selenite, 0.5 mg/ml hydrocortisone, 30 ng/ml cholera toxin A subunit, 5% fetal bovine serum (FBS), 50 mg/ml gentamicin, and 1.25 mg/ml amphotericin B. In brief, a total 2,000 single cells obtained from LEPC with or without reunion with P4 LNC, P4 CSC, and P10 LNC transfected with GFP or GFP-Pax6 were seeded on MMC-treated 3T3 fibroblast feeder layers for 10 days. The resultant clonal growth was fixed in 4% paraformaldehyde and assessed by 1% rhodamine B staining solution for marking clones for the measurement of colony-forming efficiency by calculating the percentage of the clone number divided by the total number of PCK + cells seeded that was determined by double immunostaining of PCK/Vim. Clone morphology was subdivided into holoclone, meroclone, and paraclone based on the criteria established for skin keratinocytes^49^.

### Forced expression of GFP-Pax6

The forced expression experiment was performed in P10 LNC on coated Matrigel in MESCM by adding Ad-GFP-Pax6, which is pre-packaged human adenovirus Type-5 vector (dE1/E3) expressing human enhanced GFP-Pax6 construct gene (NCBI reference sequence of Pax6 is BC011953) under the control of the cytomegalovirus (CMV) promoter or Ad-GFP, which is the empty vector with GFP promoter (both purchased from Vector Biolabs, Malvern, PA), at the MOI of 0, 4, 20, 100, 500 and 2500 for 1 to 5 days.

### Neurosphere formation

Single cells of both LNC or CSC expanded at different passages were plated at cell density of 5000/cm^2^ on anti-adhesive poly-HEMA in 12 well-plate for 6 days in neural stem cell medium (NSCM) consisting of 20 ng/ml EGF, 20 ng/ml FGF2, 2% NSCM supplement, and 1.6% methylcellulose^32^. Sphere formation was monitored by phase microscope and spheres with the size of greater than 50 μm in diameter were counted throughout the entire 12-well on day 6 by Zeiss Axio-Observer Z1 Motorized Inverted Microscope (Carl Zeiss, Thornwood, NY). The neurosphere-forming efficiency was calculated by subdividing the total number of spheres by the total number of seeded cells x 100%.

### Neuroglial differentiation

1 × 10^4^/ml of P4 or P10 LNC were seeded on 50 µg/ml poly-L-ornithine and 20 µg/ml laminin-coated or Collagen Type IV coated cover glass in 48-well plate in NSCM supplement with 0.5% N2 and 1% B27 for 2 days. For neuronal differentiation^57^, medium was then replaced to neuronal induction base medium containing DMEM/F12 (1:3) with 0.5% N2 and 1% B27 in additional to 10 ng/ml FGF2 and 20 ng/ml of BDNF (medium A) for 3 days and replaced with base medium in addition to 6.7 ng/ml FGF2 and 30 ng/ml of BDNF for another 3 days. Cell then replaced to base medium in addition to 2.5 ng/ml FGF2, 30 ng/ml BDNF, and 200 mM ascorbic acid for another 8 days. For oligodendrocyte differentiation^57^, medium then replaced with base medium containing DMEM/F12 (1:1) with 1% N2 in addition to 10 ng/ml FGF2, 10 ng/ml PDGF, and 10 μM forskolin for 4 days and then medium was replaced by the base medium in addition to 10 ng/ml FGF2, 30 ng/ml 3,3,5-triiodothyronine, and 200 μM ascorbic acid for another 7 days. For astrocyte differentiation (Thermo Scientific, Santa Clara, CA), medium was replaced by DMEM containing 1% FBS, 1% N2, and 2 mM GlutaMax for 10 days. Induction media were changed every 3–4 days. Media and supplements are listed in Supplementary Table S2.

### RNA extraction, reverse transcription and quantitative real-time PCR

Total RNAs were extracted from expanded LNC, CSC, or neurospheres on day 6 by RNeasy Mini Kit (Quiagen, Valencia, CA) according to manufacturer’s guideline and 1–2 ug of RNA extract was reverse transcribed to cDNA with reverse-transcribed using High Capacity Reverse Transcription Kit (Applied Biosystems, Foster City, CA) using primers listed in Supplementary Table S3. The resultant cDNAs were amplified by specific TaqMan gene expression assay mix and universal PCR master mix in 7300 Real Time PCR System (Applied Biosystems, Foster City, CA) with real-time RT-PCR profile consisting of 10 min of initial activation at 95 °C, followed by 40 cycles of 15 sec denaturation at 95 °C, and 1 min annealing and extension at 60 °C. The relative gene expression data were analyzed by the comparative CT method (ΔΔCT). All assays were performed in triplicate. The results were normalized by glyceraldehyde 3-phosphate dehydrogenase (GAPDH) as an internal control.

### Immunofluorescence staining

Single cells of LNC or CSC at different passages and their neurospheres with or without knockdown by forced expression of Pax6 were harvested with 0.05% trypsin and 1 mM EDTA at 37 °C for 10 min and prepared for cytospin using Cytofuge (StatSpin Inc., Norwood, MA) at 1000 rpm for 8 min. Cells were fixed with 4% formaldehyde, pH 7.0, for 15 min at room temperature, permeabilized with 0.2% Triton X-100 in PBS for 15 min and blocked with 2% bovine serum albumin (BSA) for 1 h before incubated with primary antibodies for 16 h at 4 °C. After 3 washes with PBS, the corresponding Alexa Fluor-conjugated secondary IgG (all 1:100 dilution) were incubated for 60 min and 3 washing with PBS. *A*s previously reported^2^, the method to calculate the % nuclear Pax6 positive cells was based on counting of nuclear Pax6 positive cells using AxioVision software (Carl Zeiss, Thornwood, NY) of immunofluorescence staining images with Pax6 staining and Hoechst 33342 counter nuclear staining taken by confocal microscopy. Corresponding mouse and rabbit sera were used as negative controls for the primary monoclonal and polyclonal antibodies, respectively. Detailed information about primary and secondary antibodies and agents used for immunostaining is listed in Supplementary Table S4. Neurospheres were also incubated in NSCM containing 4 µM of EthD-1 and 2 µM of Calcein AM at 37 °C for 30 min for fluorescence detected at 494/517 nm for viable and 528/617 nm for dead cells, respectively under the confocal microscope.

### Western blot

Cell lysates were extracted from P10 LNC transfected with Ad-Pax6 GFP or Ad-GFP on day 4 by cold lysis buffer containing immunoprecipitation assay buffer, protease inhibitor cocktail (100x) and 1 mM phenylmethylsulfonyl fluoride.(Sigma-Aldrich, St. Louis, MO) Total protein of the cell lysate was measured and normalized by the BCA assay (Pierce, Rockford, IL) and 5 µg of protein lysate was loaded on a 4–15% (w/v) gradient sodium dodecyl sulfate-polyacrylamide gel and transferred to nitrocellulose membrane using mini Trans-blot electrophoretic transfer apparatus (Bio-Rad, Hercules, CA). Each membrane was blocked with 5% (W/V) fat-free milk in 50 mM Tris-HCl, pH 7.5, containing 150 mM NaCl, and 0.05% Tween-20 for 1 h before incubation with specific primary antibodies in 5% (W/V) fat-free milk overnight at 4 °C follow by their respective horseradish peroxidase-conjugated secondary antibodies using antibody against Histone 3 and β-actin as the loading control. Detailed information about primary and secondary antibodies and agents used is listed in Supplementary Table S4. The immunoreactive bands were detected by Western Lightning Chemiluminescence (PerkinElmer, Waltham, MA) using an ImageQuant LAS 4000 digital imaging system (GE Healthcare Piscataway, NJ).

### Statistical analysis

All summary data were reported as mean ± SD. Significance was calculated for each group and compared with two-tailed Student’s t-test and ANOVA by Microsoft Excel (Microsoft, Redmond, WA). Test results were reported as p values, where p < 0.05 were considered statistically significant.

## Supplementary information

Supplementary

## Data Availability

All the data are available for tracking.
