# Supplementary material for: Pax 6 Controls Neural Crest Potential of Limbal Niche Cells to Support Self-Renewal of Limbal Epithelial Stem Cells
Source: Sci Rep. 2019 Jul 5;9:9763. doi: 10.1038/s41598-019-45100-7 (PMC6611810; doi:10.1038/s41598-019-45100-7)
Supplement: Supplementary file 1 — Supplementary [file 41598_2019_45100_MOESM1_ESM.docx]

**Pax 6 Controls Neural Crest Potential of Limbal Niche Cells to Support Self-Renewal of Limbal Epithelial Stem Cells**

Szu-Yu Chen^1,2^, Anny M.S. Cheng^3,4^, Yuan Zhang^1^, Ying-Ting Zhu^1^, Hua He^1^, Megha Mahabole^1^ and Scheffer C. G. Tseng^2,4^_*_

^1^ R&D Department, Tissue Tech, Inc., Miami, FL 33126 USA.

^2^ Department of Biochemistry and Molecular Biology, University of Miami Miller School of Medicine, Miami, FL33136 USA.

^3^ Department of Ophthalmology, Florida International University, Herbert Wertheim College of Medicine, FL33199, USA.

^4^ Ocular Surface Center, and Ocular Surface Research & Education Foundation, Miami, FL 33126 USA.

_*_**Correspondence**: Scheffer C. G. Tseng. R&D Department, TissueTech, Inc., 7235 Corporate Center Drive, Suite C, Miami, Florida 33126. Telephone: (305)274-1299; Fax: (305) 274-1297; E-mail: [stseng@ocularsurface.com](mailto:stseng@ocularsurface.com)

**Supplementary Information**

**Supplementary Fig. S1.** Nuclear expression of Sox10 (red) in P4 LNC. (Scale bar: 100 µm)

**Sox10**


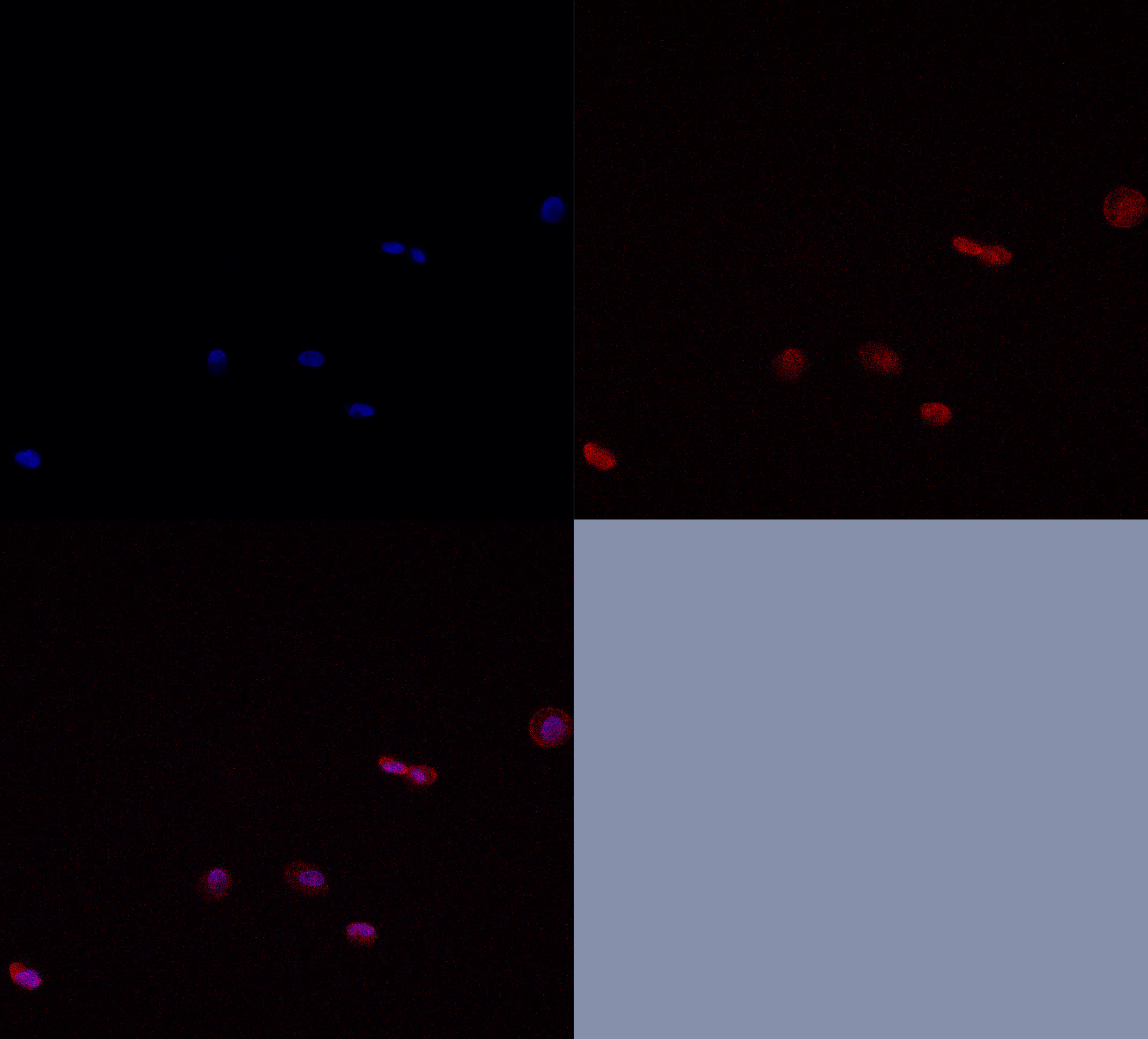

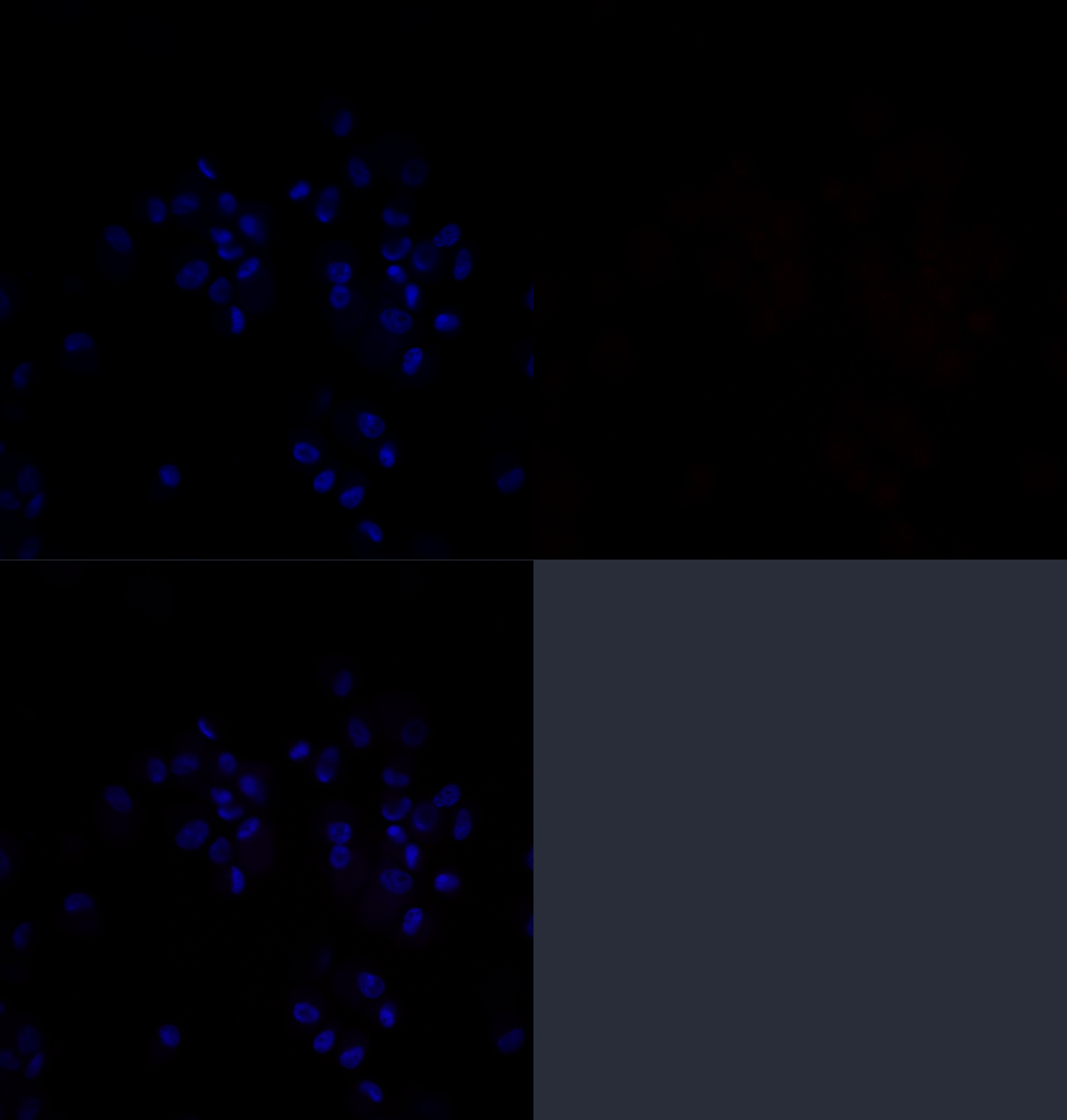


**LNC/P4**

**CSC/P4**

**Supplementary Fig. S2.** Minimal expression of transcripts of cytokeratin 12 (CK12) and cytokeratin 15 (CK15) after P2 LNC expansion.

Relative mRNA Levels

**Supplementary Fig. S3.** Reduced expression of Pax6, p75^NTR^, Musashi-1, Sox2, Nestin, FoxD3 and Msx1 of LNC isolated from Region B in serial passages P2 to P9.

Relative mRNA Levels

**Supplementary Fig. S4**. Western blot analysis used to compare the protein expression of Oct4, p75NTR, Musashi-1 and β-actin.

**β-actin Oct4**

**
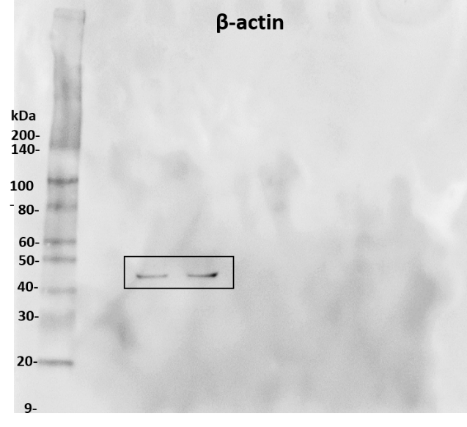

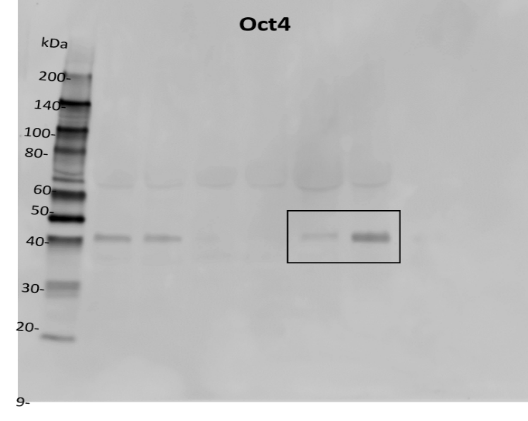
**

**p75^NTR^ Musashi-1**

**
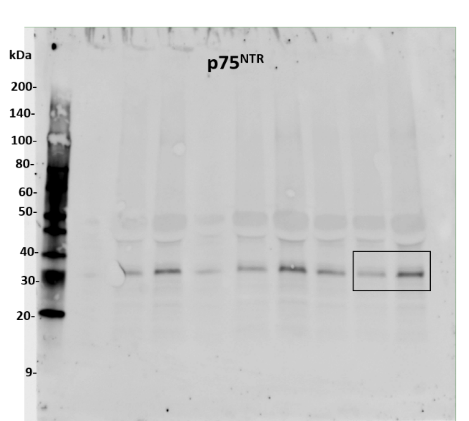

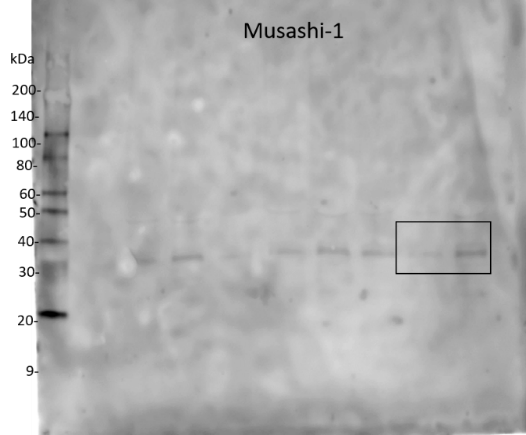
**

**Supplementary Fig. S5**. Western blot analysis used three Pax6 antibodies purchased from three different vendors to compare the protein expression of Pax6**.**

**
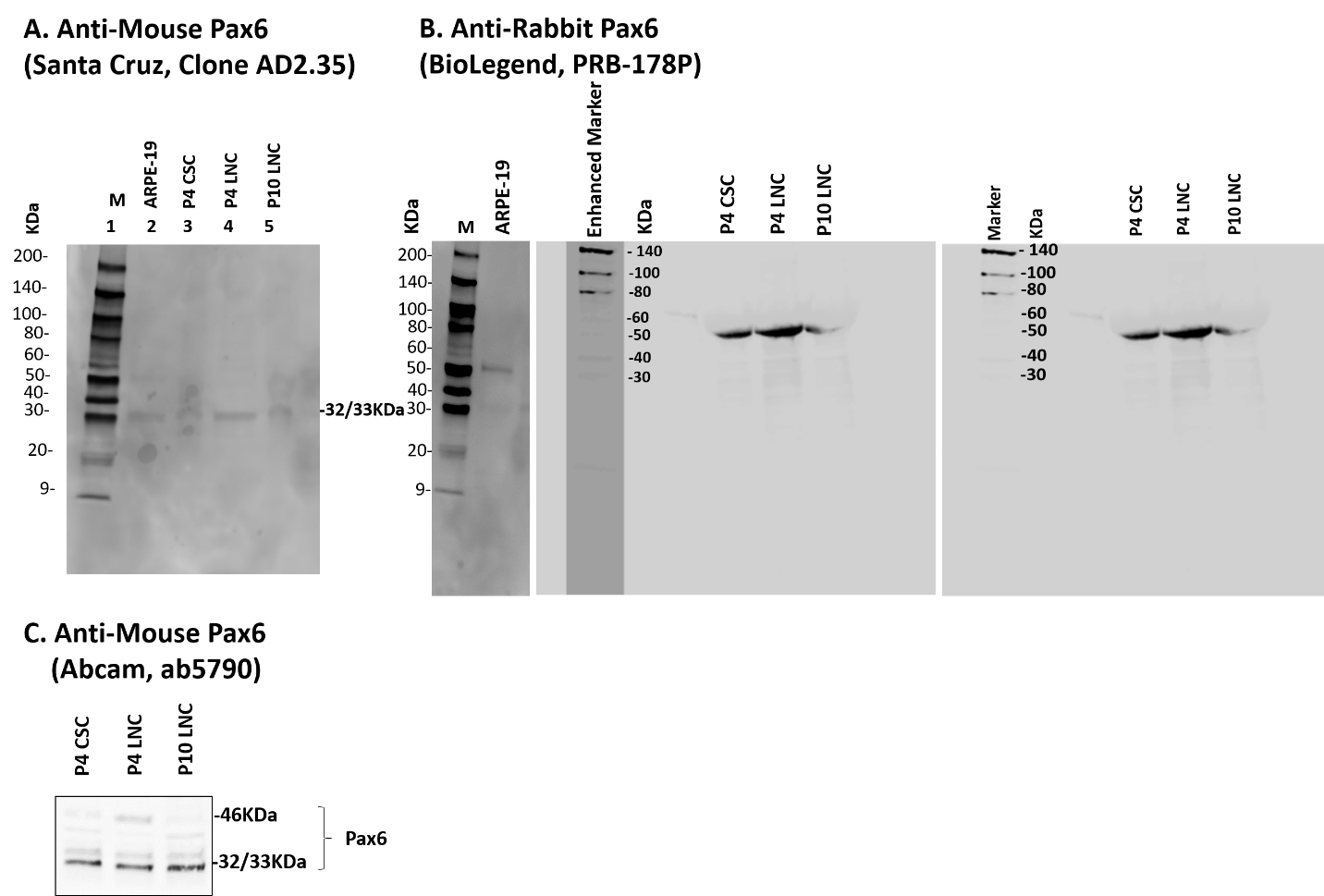
**

**D Pax6 (BioLegend, PRB-178P)**

**GFP-Pax6**

**M**

**ARPE**

**-19**

**GFP**

60-

40-

30-

50-


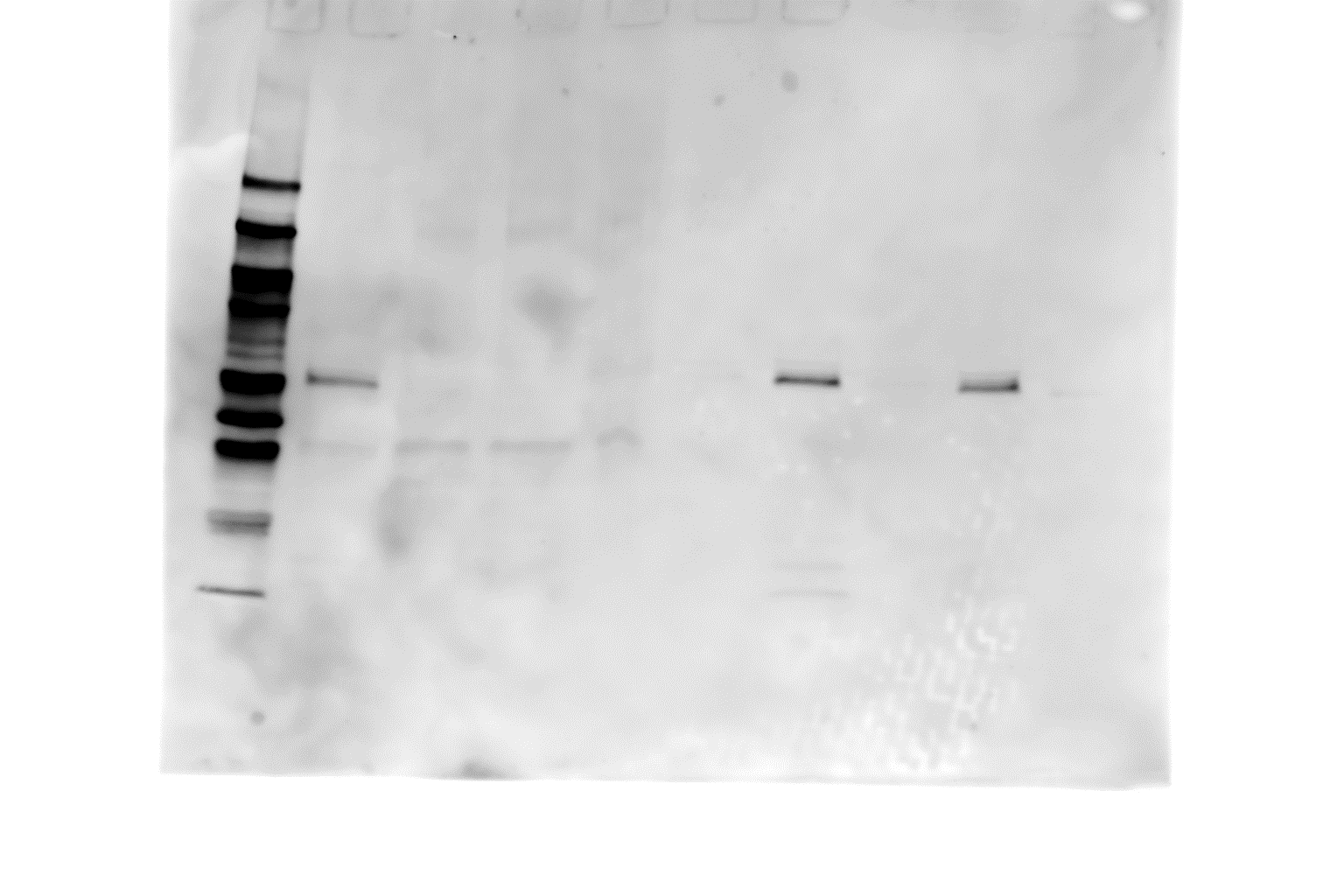


**-46KDa**

**Supplementary Fig. S6.**

The epithelial morphology of meroclone of all reunion group was characterized by immunostaining of Hoechst 33342 (A, Blue) Pax6 (B, red), CK15 (C, green) and merged (D). (Scale bar: 50 µm)


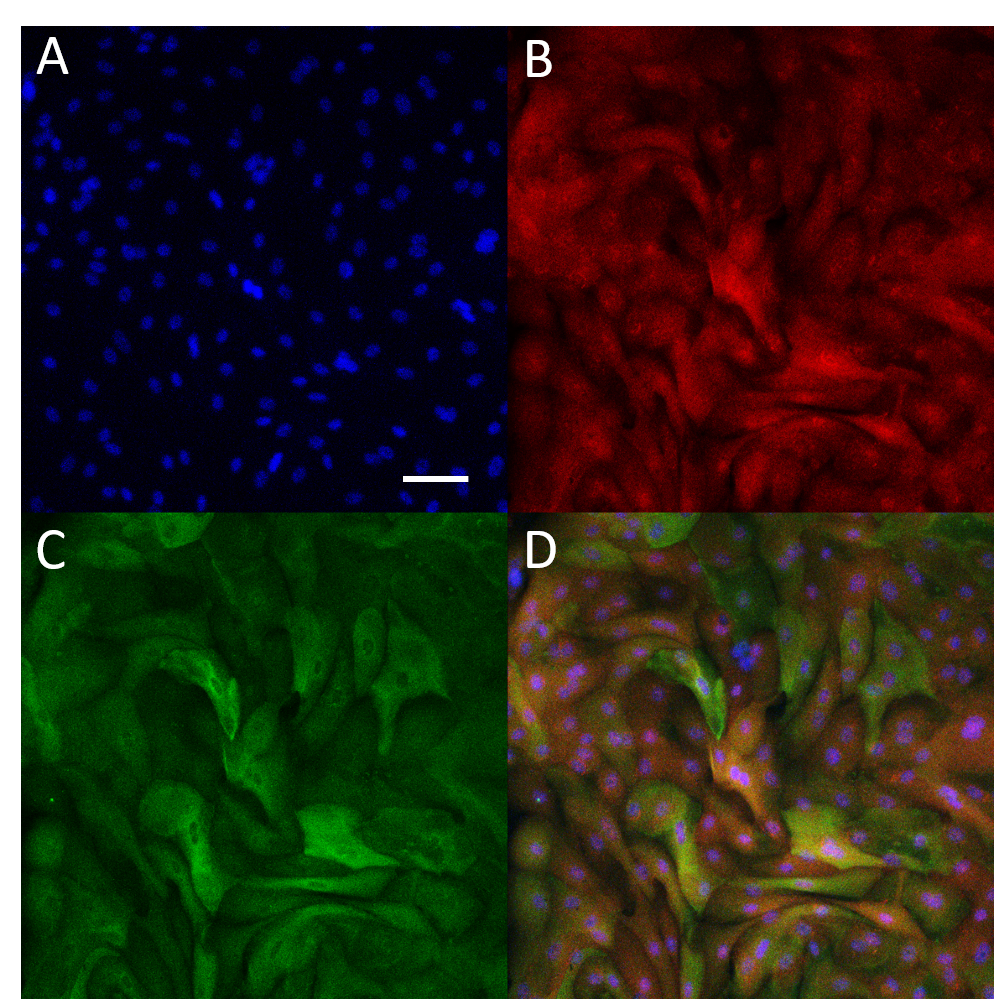


The epithelial morphology of holoclone of Adeno-GFP-Pax6 was characterized by immunostaining of Hoechst 33342 (A, Blue) Pax6 (B, red), CK15 (C, green) and merged (D). (Scale bar: 50 µm)

**
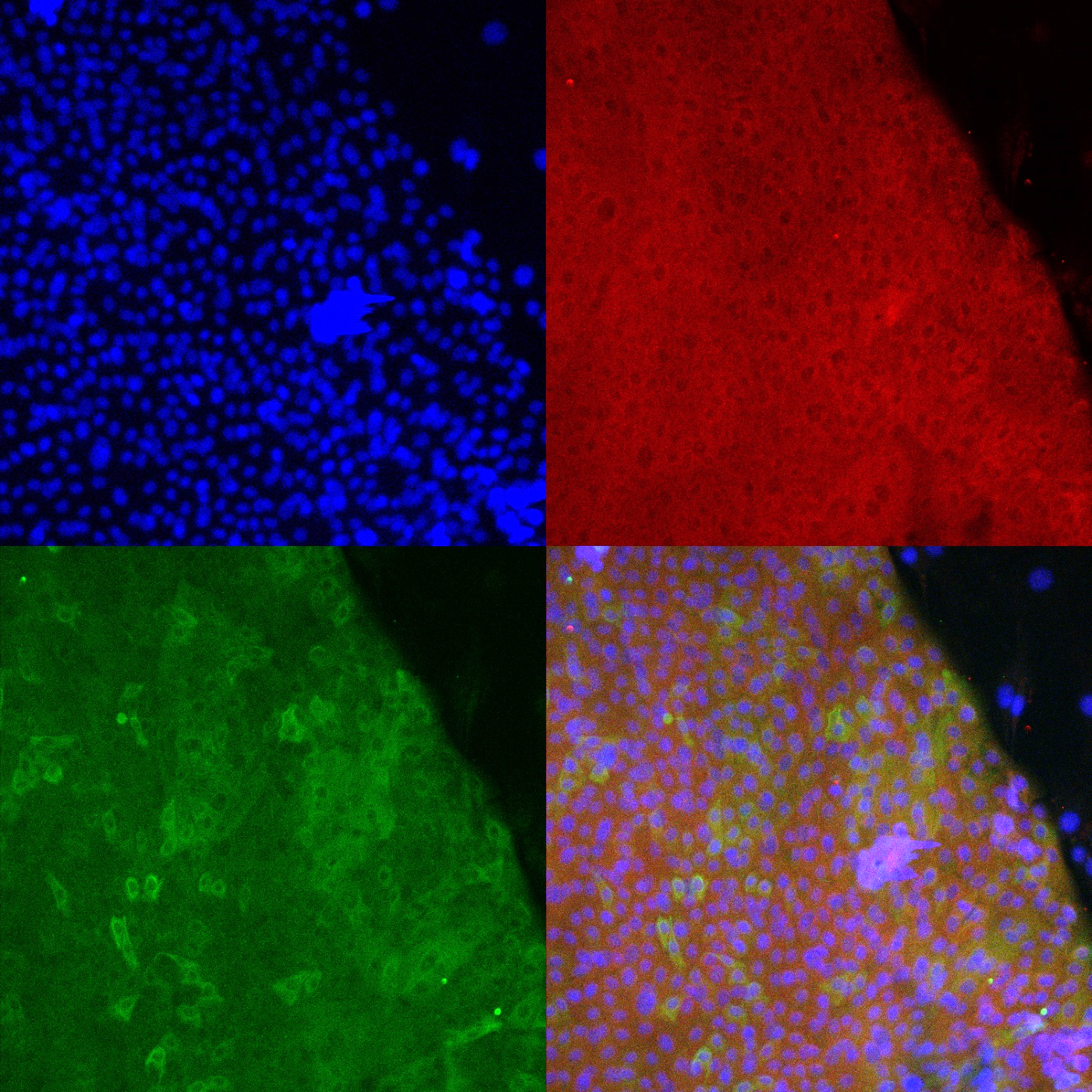
**

**Supplementary Table S1.** Material Used for Cell Isolation and Expansion.

| **Name** | **Source** | **Concentration** |
| --- | --- | --- |
| DMEM | Invitrogen, Grand Island, NY | DMEM/F-12 (1:1) |
| F-12 | Invitrogen, Grand Island, NY | DMEM/F-12 (1:1) |
| Knockout Serum Replacement | Invitrogen, Grand Island, NY | 10% |
| Human Fibroblast Growth Factor-Basic | Invitrogen, Grand Island, NY | 4 ng/ml |
| Leukemia inhibitory factor (LIF) | Invitrogen, Grand Island, NY | 10 ng/ml |
| Dimethyl Sulfoxide | Sigma-Aldrich,St. Louis, MO | 0.5% |
| L- glutamine | Invitrogen, Grand Island, NY | 1 mM |
| Insulin-Transferrin- sodium selenite media supplement | Roche,Indianapolis, IN | 5 µg/ml insulin, 5 µg/ml Transferrin, 5 ng/ml sodium selenite |
| 0.05%Trypsin-EDTA (T/E) | Invitrogen, Grand Island, NY | 0.25% and1mM |
| Fetal Bovine Serum | Atlas, Fort Collins, CO. | 100% Bovine Serum |
| StemPro NSC SFM Kit | Thermo Fischer | 50 EU/ml |
| Phosphate-Buffered Saline pH 7.4 (PBS) | Invitrogen, Grand Island, NY | 1X |
| Amphotericin B | Invitrogen, Grand Island, NY | 50µg/ml |
| Gentamicin | Invitrogen, Grand Island, NY | 1.25µg/ml |
| Dispase II | Roche,Indianapolis, IN | 10 mg/ml |
| Collagenase A | Roche,Indianapolis, IN | 2 mg/ml |
| Matrigel Basement Membrane Matrix | BD Bioscience, San Jose, CA | 100% |
| poly-2-hydroxyethyl methacrylate (pHEMA) | Sigma Aldrich, St Louis, MO | 120 mg/ml (Solubility)  1.15 g/ml (density) |
| Methylcellulose(15c Ps) | Sigma-Aldrich, St Louis, MO | 1.6%  Viscocity: 3,500-5,600 cP |
| Paraformaldehyde | Sigma-Aldrich, St Louis, MO | 4% |
| Methanol | Sigma-Aldrich, St Louis, MO | 100% |
| Bovine serum albumin (BSA) | Sigma-Aldrich, St Louis, MO | 40 mg/ml |
| Triton X-100 | Sigma-Aldrich, St Louis, MO | 0.2-0.9 mM |
| RNeasy Mini Kit | Qiagen, Valencia, CA | Capacity of 100 μg RNA |
| Live/Dead viability/cytotoxicity kit | Molecular Probes, Eugene, OR | N/A |
| VECTASHIELD Anti-fade mounting medium | Vector Lab, Burlingame, CA | Unit Size:10 ml |

**Supplementary Table S2.** Primary and Secondary Antibodies used for Immunofluorescence staining.

| **Primary Antibodies** | | | |
| --- | --- | --- | --- |
| **Antibody** | **Supplier/Catalog (City, State)** | **Source** | **Dilution** |
| β-actin | Santa Cruz/SC-47778 (Santa Cruz, CA) | Chicken | 1:1000 |
| Vimenin | Abcam/ab11256-20 (Cambridge MA) | Rabbit | 1:100 |
| Sox2 [57CT23.3.4] | Abcam/MAB2018 (Cambridge MA) | Rabbit | 1:100 |
| Musashi-1 | Abcam/ab52865 (Cambridge MA) | Rabbit | 1:100 |
| Pax6 (Wu et al nature commun, 2016) | Abcam/ab5790 (Cambridge MA) | Rabbit | 1:100 |
| Pax6(Osumi et al Development 1997) | Biolegend/901301 (Cambridge MA) | Rabbit | 1:100 |
| Nestin | Abcam/ab22035 (Cambridge MA) | mouse | 1:100 |
| P75^NTR^ | Abcam/ab52987 (Cambridge MA) | Rabbit | 1:100 |
| PDGFRβ | Abcam/ab3257 (Cambridge MA) | Rabbit | 1:100 |
| a-SMA | Dako/M0851 (Carpinteria, CA) | Mouse | 1:100 |
| CD31 | Cell Signaling/3528S (Danvers, MA) | Mouse | 1:100 |
| Sox10 | Cell Signaling/89356T(Danvers, MA) | Rabbit | 1:100 |
| OCT4 (POU5F1) | EMD Millipore/MAB4401 (Billerica, MA) | Mouse | 1:100 |
| Neurofilament M | EMD Millipore/ AB1987 MAB4401 (Billerica, MA) | Rabbit | 1:100 |
| β-III Tubulin | R&D Systems/ MAB1195 (Minneapolis, MN) | Mouse | 1:100 |
| O4 | R&D Systems/MAB1326 (Minneapolis, MN) | Mouse | 1:100 |
| Ki67 | R&D Systems/AF7617 (Minneapolis, MN) | Sheep | 1:100 |
| GFAP | EMD Millipore/ AB5541 MAB4401 (Billerica, MA) | Chicken | 1:100 |
| p63α | Cell Signaling/4892(Danvers, MA) | Rabbit | 1:100 |
| Cytokeratin 15 | Biolegend/ PCK-15 (Cambridge MA) | Chicken | 1:100 |
| Cytokeratin (AE1/AE3) | Dako M3515 (Carpinteria, CA) | Mouse | 1:50 |
| Keratin 12 | Santa Cruz/sc-17099 (Santa Cruz, CA) | Goat | 1:50 |
| Rabbit IgG | Dako/X0902 | - | 1:100 |
| Mouse IgG | Dako/X0910 | - | 1:100 |
| Hoechst 33342 | Sigma-Aldrich/B2261 (St Louis, MO) | N/A | 1:500 |
|  |  |  |  |
| **Secondary Antibodies** | | | |
| **Antibody** | **Supplier** | **Source** | **Dilution** |
| Alexa Fluor 488 Anti-Goat | Thermo Fisher Scientific (Indianapolis, IN) | Donkey | 1: 100 |
| Alexa Fluor 633 Anti-Goat | Thermo Fisher Scientific (Indianapolis, IN) | Donkey | 1: 100 |
| Alexa Fluor 488 Anti-Mouse | Thermo Fisher Scientific (Indianapolis, IN) | Donkey | 1: 100 |
| Alexa Fluor 555 Anti-Rabbit | Thermo Fisher Scientific (Indianapolis, IN) | Donkey | 1: 100 |
| Polyclonal Rabbit Anti-Mouse IgG/HRP | Dako (Carpinteria,CA) | Rabbit | 1:1000 |
| Polyclonal Swine Anti-Rabbit IgG/HRP | Dako( Carpinteria, CA) | Donkey | 1:1000 |
| Polyclonal Rabbit Anti-Goat IgG/HRP | Dako (Carpinteria, CA) | Rabbit | 1:1000 |

**Supplementary Table S3.** Primer and Probe Sequence Use for Quantitative Real-Time PCR.

| **Assay ID and Probes Sequence** | | | |
| --- | --- | --- | --- |
| **Gene Name** | **Assay ID (TagMan Expression Assay)** | **Uni ID** | **Product Length** |
| GAPDH | Hs02758991_g1 | Hs.598320 | 93 |
| α-SMA | Hs00426835_g1 | Hs.500483 | 105 |
| CD31 | Hs00169777_m1 | Hs.376675 | 65 |
| CD73 | Hs01573922_m1 | Hs.153952 | 64 |
| CD105 | Hs00923996_m1 | Hs.76753 | 64 |
| FLK-1 (VEGFR2) | Hs00911700_m1 | Hs.479756 | 83 |
| FoxD3 | Hs00255287_s1 | Hs.546573 | 78 |
| Musashi1 | Hs01045894_m1 | Hs. 158311 | 67 |
| Msx1 | Hs00427183_m1 | Hs.424414 | 144 |
| Nanog | Hs02387400_g1 | Hs.635882 | 109 |
| Nestin | Hs00707120_s1 | Hs. 527971 | 81 |
| Oct4 | Hs00999632_g1 | Hs.249184 | 77 |
| Pax6 | Hs00240871_m1 | Hs. 270303 | 76 |
| PDGFRβ | Hs01019589_m1 | Hs.509067 | 62 |
| P75NTR | Hs00609977_m1 | Hs. 415768 | 140 |
| Sox2 | Hs01053049_s1 | Hs.518438 | 91 |
| Sox10 | Hs00366918_m1 | Hs. 376984 | 102 |
| TFAP2α | Hs00900330_m1 | Hs. 467125 | 69 |
| TFAP2β | Hs01560931_m1 | Hs. 33102 | 67 |
| ΔNp63α | Forward: GGCTGTTCATCATGTCTGGACTATT Reverse: CATCCATGGAGTAATGCTCAATCTGA | N/A | 78 |
| CK15 | Hs00267035_m1 | Hs.654570 | 81 |
| CK12 | Hs01057907_m1 | Hs.66739 | 71 |
|  |  |  |  |

**Supplementary Table S4.** Neural Differentiation Medium Use.

| **Media** | **Neuron Differentiation** | **Oligodendrocyte Differentiation** | **Astrocyte Differentiation** |
| --- | --- | --- | --- |
| **Base Medium** | DMEM/F12 (1: 3) | DMEM/F12 (1: 1) | DMEM |
| **Serum** | - | - | 1%FBS |
| **Substrate** | poly-L-ornithine and laminin-coated | Collagen Type IV | Collagen Type IV |
| **Supplements** | Medium A   - N2 (0.5%) - B27 (1%) - FGF2 (10ng/ml) - BDNF (20ng/ml)   Medium B   - N2 (0.5%) - B27 (1% ) - FGF2 (6.7ng/ml) - BDNF (30ng/ml)   Medium C   - N2 (0.5%) - B27 (1% ) - FGF2 (2.5ng/ml) - BDNF (30ng/ml) - 200mM ascorbic acid | Medium A   - 1% N2 - 10ng/ml FGF2 - 10uM forskolin - 10ng/ml PDGF-AA   Medium B   - 1% N2 - 10ng/ml FGF2 - 30 ng/ml 3,3,5 triiodothyronin (T3) hormone - 200mM ascorbic acid | - N2 (1%) - B27 (2%) - 2mM GlutaMax |
| **Length of Culture (Days)** | Medium A: 3  Medium B: 3  Medium C: 10 | Medium A: 4  Medium B:7 | 7 |

| **Name** | **Supplier/Catalog** | **Source** | **Concentration** |
| --- | --- | --- | --- |
| StemPro^TM^ Neural Stem Cells Serum-Free Expansion Medium (NSCM) | Thermos Fisher/A1050901 | N/A | N/A |
| Recombinant FGF | Thermo Fisher/PHG0024 | Human | 2.5-10ng/ml |
| BDNF | Sigma-Aldrich/B3795 | Human | 20-30 ng/ml |
| L-Ascorbic Acid-2-phosphate | Sigma-Aldrich/A8960 | N/A | 200mM |
| Poly-L-Ornithine hydrobromide | Sigma-Aldrich/P3655 | N/A | 50ug/ml |
| Forskolin | Sigma-Aldrich/F6886-10MG | N/A | 10uM |
| PDGF-AA | Stem Cell Technologies/78095 | Human | 10ng/ml |
| Triiodothyronin hormone (T3) | Sigma-Aldrich/T6397 | Human | 30ng/ml |
| Collagen IV | Millipore Sigma/234154-100UG | Human | 50 ug/ml |
| Laminin | Thermo Fisher/23017-015 | Mouse | 20 ug/ml |
| N2/B27/Heparin (StemPro™) | ThermoFisher/A1050801 | N/A | 2% |
| GlutaMax | ThermoFisher/ 35050-061 | N/A | 2mM |
| B-27 | Thermo Fisher/17504044 | N/A | 1-2% |
| N2 | Thermo Fisher/1370701 | N/A | 0.5-1% |
